# Optotracing for selective fluorescence-based detection, visualization and quantification of live *S. aureus* in real-time

**DOI:** 10.1038/s41522-020-00150-y

**Published:** 2020-10-09

**Authors:** Karen Butina, Ana Tomac, Ferdinand X. Choong, Hamid Shirani, K. Peter R. Nilsson, Susanne Löffler, Agneta Richter-Dahlfors

**Affiliations:** 1 AIMES-Center for the Advancement of Integrated Medical and Engineering Sciences at Karolinska Institutet and KTH Royal Institute of Technology, Stockholm, Sweden; 2 Department of Neuroscience, Karolinska Institutet, SE-171 77 Stockholm, Sweden; 3 Department of Chemistry, IFM, Linköping University, SE-581 83 Linköping, Sweden

**Keywords:** Bacteria, Biological techniques, Pathogens

## Abstract

Methods for bacterial detection are needed to advance the infection research and diagnostics. Based on conformation-sensitive fluorescent tracer molecules, optotracing was recently established for dynamic detection and visualization of structural amyloids and polysaccharides in the biofilm matrix of gram-negative bacteria. Here, we extend the use of optotracing for detection of gram-positive bacteria, focussing on the clinically relevant opportunistic human pathogen *Staphylococcus aureus*. We identify a donor-acceptor-donor-type optotracer, whose binding-induced fluorescence enables real-time detection, quantification, and visualization of *S. aureus* in monoculture and when mixed with gram-negative *Salmonella* Enteritidis. An algorithm-based automated high-throughput screen of 1920 *S. aureus* transposon mutants recognized the cell envelope as the binding target, which was corroborated by super-resolution microscopy of bacterial cells and spectroscopic analysis of purified cell wall components. The binding event was essentially governed by hydrophobic interactions, which permitted custom-designed tuning of the binding selectivity towards *S. aureus* versus *Enterococcus faecalis* by appropriate selection of buffer conditions. Collectively this work demonstrates optotracing as an enabling technology relevant for any field of basic and applied research, where visualization and detection of *S. aureus* is needed.

## Introduction

The capability to detect and identify the bacteria is essential in many sectors of our society. Effective treatment of bacterial infections requires methods to accurately and quickly identify the causative pathogen. Such methods are not only required in the health care sector, but are similarly important for industrial quality control, i.e., the food and pharma industries, where microbial contamination must be avoided. There is also a need in the research community for nontoxic stains that allow real-time monitoring of live bacteria to understand infection dynamics in vitro and in vivo.

While novel technologies for bacterial identification are under development in research laboratories, current approaches in clinical settings typically rely on culture-based techniques^1,2^. This time-consuming step is necessary to increase the bacterial numbers to levels detectable by optical density-based recordings prior to analysis. Gram stain offers a simple method for preliminary identification of bacteria based on the peptidoglycan (PGN) layer in its cell wall. This staining technique is however associated with a discrepancy of 5% compared to culture results, partly explained by laborious sample preparation, the manual nature of the staining process, and subjective assessment of results^3^.

Synthetically designed conjugated polyelectrolytes and oligoelectrolytes and oligomers were recently developed for imaging bacteria^4,5^. These cationic molecules bind the negatively charged cell envelope of gram-positive and gram-negative bacteria via electrostatic interactions^4–6^. Cationic agents are, however, known to disrupt the bacterial cell envelope, which explains the bactericidal effects commonly observed by those ligands^6^. To contrast this, optotracing was developed as a nontoxic alternative for detection of polymeric substances. Optotracers are small, chemically well-defined, anionic fluorescent tracer molecules based on luminescent conjugated oligothiophenes, which via electrostatic interactions bind their target molecules^7,8^. Binding to specific biological targets can be controlled by chemical design, enabling tailored charge distribution along the conjugated thiophene backbone. By varying the length and molecular composition of the conjugated backbone, the photo-physical characteristics of optotracers can be tuned^9,10^. Optotracers have been employed for spectral discrimination of amyloid morphotypes^11^. Interaction with amyloids leads to a characteristic flattening of the molecular backbone and a more effective conjugation, which is detected by a red-shift in the fluorescence excitation and increased fluorescence emission intensity. In addition, a new generation of optotracers has been developed that presents an on-like switching of fluorescence upon binding to its target^10^.

The amyloid curli fimbriae and cellulose are important components of the extracellular matrix (ECM) produced by *Escherichia coli* (*E. coli*) and *Salmonella enterica* serovars Enteritidis (*S*. Enteritidis) and Typhimurium (*S*. Typhimurium) when entering the biofilm lifestyle^12,13^. We developed optotracing as the hitherto only method that directly traces ECM production in situ in real-time^14^. The structural change optotracers undergo when binding to a target is instantly translated to altered intensity and spectral properties of emitted light. When used as nontoxic medium additives, photostable optotracers incorporated into the growing biofilm emit target-specific optical signals as the target is produced, thus allowing true real-time analysis. Optotracing also enabled the development of a diagnostic assay for biofilm-related urinary tract infections by detection of native cellulose produced by uropathogenic *E. coli* (UPEC) in urine from infected patients^15^. We expanded optotracing to become a versatile method for nondisruptive analysis of polysaccharides^16^. The donor–acceptor–donor (D–A–D)-type electronic structure of the optotracers’ conjugated backbone increased the spectral separation of bound optotracers. A remarkable sensitivity to the glucan stereochemistry was observed, with β-configured cellulose readily differentiated from α-configured glucans^16^.

Here, we extend our use of optotracers for polysaccharide reporting by investigating their potential for detection of *Staphylococcus aureus*. Similar to cellulose, glycan strands in the cell wall of gram-positive bacteria are composed of β-(1 → 4)-linked repeating disaccharide units, suggesting that the glycan backbone may possess binding sites for optotracers. Catering to the need for rapid and automated methods, we explore the use of optotracing and algorithm-based analytics for easy bacterial detection and identification.

## Results

### Optotracing for detection and quantification of *Staphylococci*

**Fig. 1:**
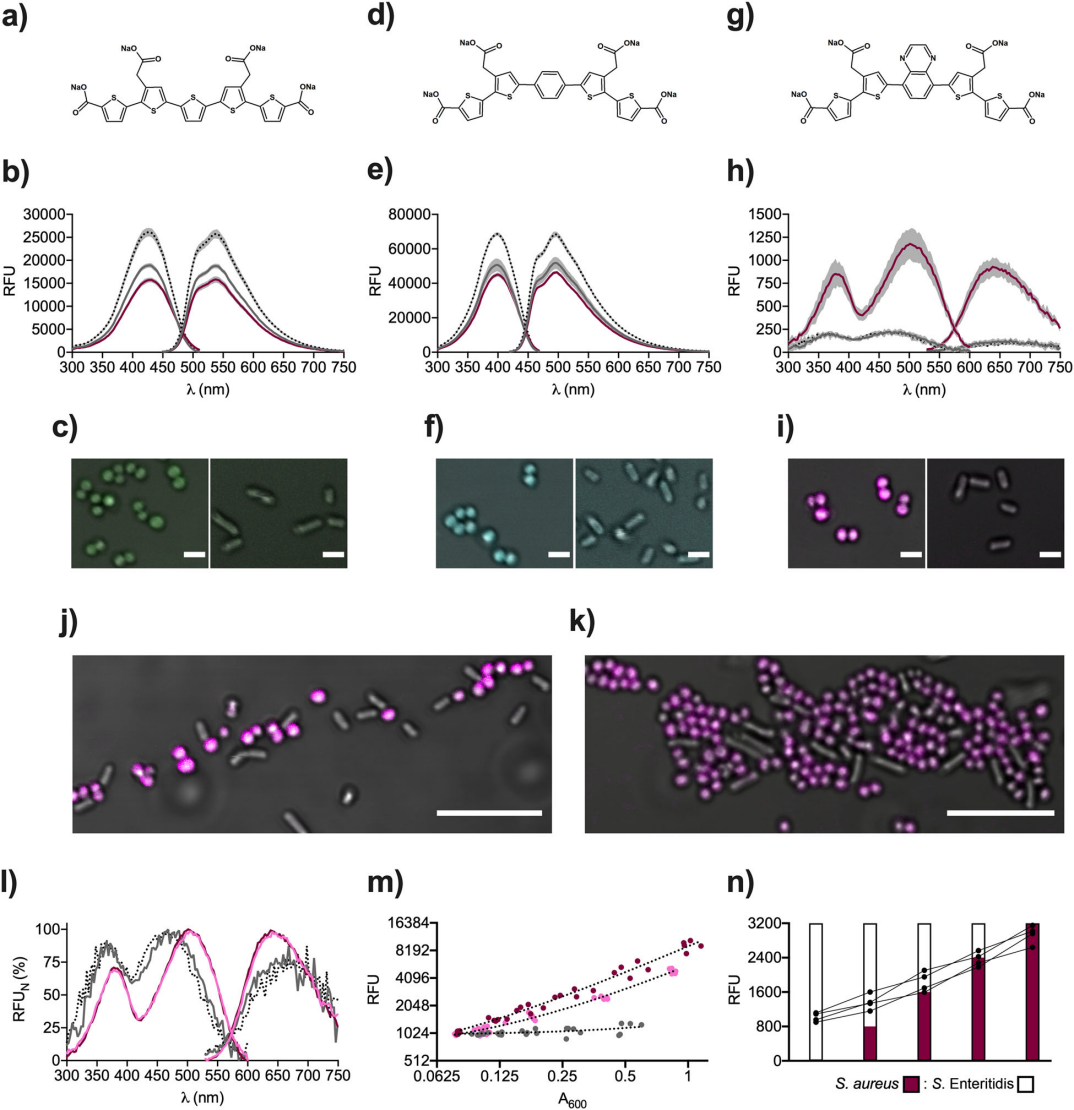
Fluorescence spectroscopy and microscopy using optotracers. **a**, **d**, **g** The chemical structures of **a** HS-84, **d** HS-163, and **g** HS-167. **b**, **e**, **h** Specplots of **b** HS-84, **e** HS-163, and **h** HS-167 in PBS (black dotted line) and when mixed with *S. aureus* (magenta) and *S*. Enteritidis (gray). Lines = mean values from *n* = 3–5, shaded areas = ± SD. **c**, **f**, **i** Merged transmitted light and pseudocolored confocal images of *S. aureus* (left) and *S*. Enteritidis (right) mixed with **c** HS-84 (green), **f** HS-163 (cyan), **i** HS-167 (magenta), in PBS. Scale bar = 2 µm. Corresponding full-size images are shown in (Fig. S1c–e**)**. **j**, **k** Merged transmitted light and pseudocolored confocal images of HS-167 (magenta) in a mixed sample of **j** *S. aureus* and *S*. Enteritidis, and **k** *S. epidermidis* and *E. coli*. Scale bar = 10 µm. **l** Normalized specplot showing normalized RFU (RFU_N_) of HS-167 in PBS (dotted line) with added *S. aureus* (magenta), *S. epidermidis* (pink) and *S*. Enteritidis (gray). Lines = mean values from *n* = 5. **m** Fluorescence intensity (RFU) from HS-167, recorded at Ex. 507 nm and Em. 625 nm, in samples of *S. aureus* (magenta), *S. epidermidis* (pink) and *S*. Enteritidis (gray) at different cell densities (A_600_). *n* = 5, all experimental points are shown from each experiment. Dotted lines show linear fit. **n** Fluorescence intensity (RFU) from HS-167, recorded at Ex. 507 nm and Em. 625 nm, in mixes containing defined ratios of *S. aureus* and *S*. Enteritidis. Black dots show experimental points (*n* = 4) connected by lines. Bars show the proportion of *S. aureus* (magenta) and *S*. Enteritidis (white) in each sample.

To identify an optotracer capable of detecting *Staphylococci*, we used thiophene-based pentamers with different planarity and conjugation length, but equal charge distribution from anionic side groups. We evaluated the feasibility of optotracing for detection of *S. aureus* (8325-4) by characterizing the photophysical properties of optotracers in the presence and absence of bacteria, using gram-negative *S*. Enteritidis (3934) for comparison (Table S1). The optotracer HS-84 (Fig. 1a) was added to bacterial cells resuspended in phosphate-buffered saline (PBS), aliquots were transferred into 96-well plates, and excitation and emission spectra of each sample were recorded in a plate reader. Recordings of HS-84 in PBS defined the optotracer’s spectrum in the unbound state. Analysis of data in a spectral plot (spec-plot) showed broad excitation and emission ranges of unbound HS-84 with maximum excitation (Ex. *λ*_max_) at 426 nm, and maximum emission (Em. *λ*_max_) at 539 nm (Fig. 1b). A similar pattern appeared for HS-84 mixed with *S. aureus* (Ex. *λ*_max_ 430 nm, Em. *λ*_max_ 537 nm) and *S*. Enteritidis (Ex. *λ*_max_ 428 nm, Em. *λ*_max_ 538 nm), albeit the fluorescence intensities were reduced. The spectral similarities became even more apparent when we analysed data in a normalized spec-plot, in which we assigned 0% to the lowest and 100% to the highest relative fluorescence unit (RFU) in each sample (Fig S1a). The lack of spectral shifts in the presence versus absence of bacteria may result from an inability of HS-84 to bind bacteria. Alternatively, binding occurred without any conformational change of the optotracer. To address this, we analysed whether bacteria mixed with HS-84 showed fluorescence from potentially bound optotracers by imaging in a confocal microscope using 405 nm excitation laser, collecting emitted light at 490–590 nm. *S. aureus* were observed as weakly fluorescent, while no signal was detected from *S*. Enteritidis (Fig. 1c), suggesting that HS-84 binds *S. aureus* but not *S*. Enteritidis. However, the absence of a spectral shift upon binding to *S. aureus* makes this optotracer suboptimal for detection of *S. aureus* in solution.

Next, we tested HS-163 whose photophysical properties differ from HS-84 as the molecular structure involves a central phenyl moiety that disrupts the planarity and conjugation length of this optotracer (Fig. 1d). The suitability of HS-163 for bacterial detection was tested by optotracing in the absence and presence of bacteria. Spec-plot analysis showed broad excitation and emission spectra of unbound HS-163 with Ex. *λ*_max_ 399 nm and Em. *λ*_max_ 496 nm (Fig. 1e). Similar spectra were generated from HS-163 mixed with *S. aureus* (Ex. *λ*_max_ 399 nm, Em. *λ*_max_ 498 nm) and *S*. Enteritidis (Ex. *λ*_max_ 399 nm, Em. *λ*_max_ 495 nm), however, with reduced fluorescence intensity. Analysing data in a normalized spec-plot confirmed the absence of spectral shifts (Fig. S1b). Imaging by confocal microscopy showed fluorescence of *S. aureus* mixed with HS-163, while no signal was detected from *S*. Enteritidis (Fig. 1f). Thus, similar to HS-84, the limited changes in fluorescence properties of HS-163 make this optotracer unsuitable for detection of *S. aureus*.

To test if a planar optotracer with extended photophysical characteristics is better for detection of *S. aureus* we used HS-167, a D–A–D type optotracer containing a central quinoxaline motif (Fig. 1g). Optotracing of HS-167 in PBS showed very low fluorescence intensity of the two excitation peaks (Ex1. *λ*_max_ 367 nm, Ex2. *λ*_max_ 460 nm), which represent the π–π* and the D–A transitions typical for this class of molecules^10^ (Fig. 1h). The “on” like switching of fluorescence from HS-167 bound to a target became apparent when mixing with *S. aureus*, since the excitation and emission spectra demonstrated an approximately 5-fold increased intensity. In contrast, spectra from HS-167 mixed with *S*. Enteritidis fully overlapped with the low-intensity spectra from unbound HS-167. As we imaged bacteria mixed with HS-167, confocal microscopy showed bright fluorescence from *S. aureus*, but no signal from *S*. Enteritidis (Fig. 1i). This prompted us to test the method in a mixed species sample containing *S. aureus* and *S*. Enteritidis. HS-167 was added to a mix of bacteria in PBS, and the sample was imaged by confocal microscopy. Brightly fluorescent coccoid *S. aureus* cells were clearly distinguished from nonfluorescent, rod-shaped *S*. Enteritidis (Fig. 1j). To investigate if HS-167 also binds other species of *Staphylococci*, we analysed a mixed species sample composed of *Staphylococcus epidermidis* (*S. epidermidis*, RP62A) and *E. coli* (ATCC 25922). Confocal microscopy showed brightly fluorescent coccoid *S. epidermidis*, while rod-shaped *E. coli* cells remained unstained (Fig. 1k). Collectively, this identifies HS-167 as a suitable optotracer for detection of *S. aureus* and *S. epidermidis*, offering a method that differentiates them from gram-negative *S*. Enteritidis and *E. coli* in single and mixed-species samples.

The principles of selective HS-167 binding and staining of *Staphylococci* presumably depend on photophysical changes of HS-167 interacting with bacteria. Spec-plot analysis showed that peak excitation and emission of HS-167 mixed with *S*. Enteritidis (Ex. *λ*_max_ 467 nm, Em. *λ*_max_ 676 nm) closely resembled the unbound form of HS-167 (Ex. *λ*_max_ 460 nm, Em. *λ*_max_ 688 nm) (Fig. 1l). In contrast, binding to *S. aureus* and *S. epidermidis* induced a red shift in peak excitation (Ex. *λ*_max_ 505 nm) and blue shifts in peak emissions (*S. aureus* Em. *λ*_max_ 645 nm, *S. epidermidis* Em. *λ*_max_ 647 nm) compared to unbound HS-167. The reduced Stokes shift and remarkable increase in fluorescence intensity from HS-167 binding to *Staphylococci* thus define the photophysical features making HS-167 an excellent candidate for binding to and staining of *Staphylococci*.

Having developed a selective optical method for detection of *Staphylococci*, we investigated its suitability for quantification of bacterial cells using a fluorescence read-out. We added HS-167 to serial dilutions of bacteria and transferred the mixtures to 96-well plates. Using a plate reader, we measured bacterial density by recording absorbance (AU) at 600 nm (A_600_), and fluorescence emitted at 625 nm when excited at 507 nm. By plotting RFU as a function of A_600_, we observed a linear correlation between fluorescence and A_600_ for *S. aureus* and *S. epidermidis* (Fig. 1m). In contrast, no fluorescence increase was observed for *S*. Enteritidis, further validating the lack of binding of HS-167 to gram-negative *Salmonella*. Fitting the data with linear regression showed that the slope for *S. aureus* (8861 ± 1406 RFU/AU) was 1.75× higher than that of *S. epidermidis* (5043 ± 473 RFU/AU). The slope differences may originate from varying abundance and/or accesibility of the binding target(s), or from minor structural differences in the binding target(s) on *S. aureus* and *S. epidermidis*. This suggests a potential of using slope analysis for species discrimination within the *Staphylococcus* genus. The quantification of *Staphylococci* by optotracing was next extended to investigate if the relative proportion of *S. aureus* in mixed cultures can be identified. Mixed species samples with defined ratios of *S. aureus* and *S*. Enteritidis were prepared and HS-167 was added. The fluorescence intensity increased as the proportion of *S. aureus* increased, independent of the presence of gram-negative *S*. Enteritidis (Fig. 1n). Taken together, this defines HS-167 as prime optotracer for detection and quantification of *Staphylococci*, equally suited for spectroscopic analysis and fluorescence microscopy.

### Adapting optotracing for automated high-throughput analysis

To identify the binding target of HS-167 on *S. aureus*, we aimed to screen a sequence defined transposon (Tn) library. The large library (≈2000 strains) prompted us to develop a workflow that enabled automated, high-throughput optotracing analysis based on real-time recordings in live bacterial cultures. Growth of *S. aureus* in the standard medium Tryptic Soy Broth (TSB) is associated with acidification, which may cause a problem as low pH is known to influence the photophysical properties of anionic optotracers^16,17^. To analyze if lowered pH affects the emitted fluorescence of unbound HS-167, we added HS-167 to standard (pH 7.1) and acidified (pH 5.1) TSB. Fluorescence recordings showed a 2-fold intensity increase at lower pH (Fig. S2a), suggesting a need to buffer the growth medium to preserve a stable baseline of HS-167 fluorescence during cultivation. We thus cultured *S. aureus* in regular and buffered TSB (bTSB) and collected samples for OD_600_ and pH measurements along with plating of colony forming units (CFU). During exponential growth, pH of the culture in bTSB remained >7.0, while pH in regular TSB dropped below 7.0 already in early exponential phase (Fig. S2b). However, no differences in the growth rate or viability of *S. aureus* were observed (Fig. S2c, d). In the stationary phase, a limited drop of 0.6 ± 0.02 units was observed in bTSB compared to 1.38 ± 0.17 units in regular TSB (Fig. S2b). In silico analysis showed a uniform charge distribution on HS-167 at pH ≥ 7 but not below (Fig. S2e), which may explain the 2-fold increase in fluorescence intensity observed at lower pH. Collectively, these experiments prompted us to use bTSB and to focus on the exponential growth phase in order to avoid pH-induced artefacts.

**Fig. 2:**
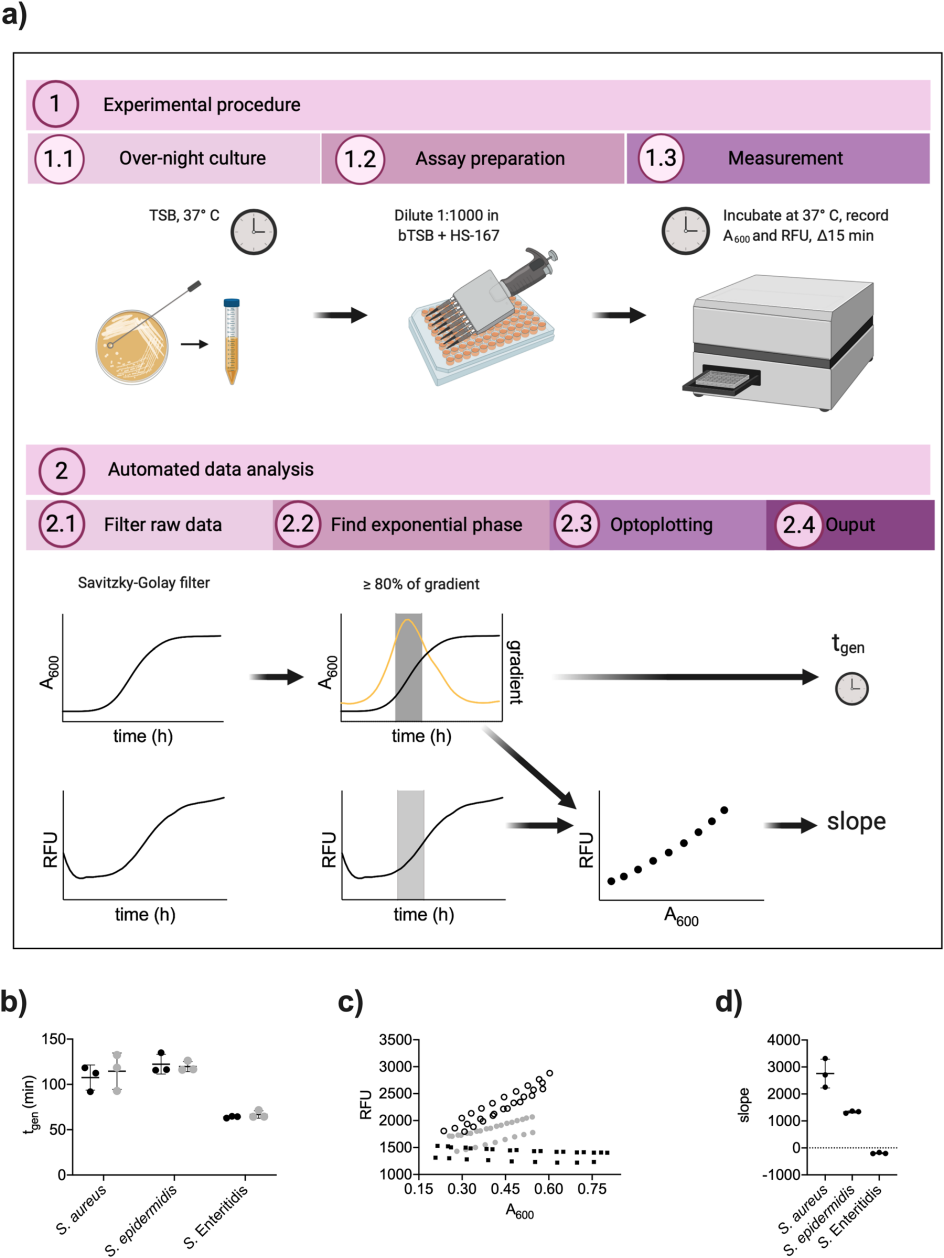
High-throughput optotracing for real-time monitoring of live bacterial cultures. **a** The workflow of automated, high-throughput optotracing analysis based on real-time recordings in live bacterial cultures. The workflow consists of: 1. Experimental procedure and 2. Automated data analysis. The figure was created with Biorender.com **b** Generation time for *S. aureus, S. epidermidis* and *S*. Enteritidis in bTSB with (gray) and without (black) HS-167. Data were analysed by *t*-test (df = 4, *p* = 0.64 for *S. aureus*, *p* = 0.73 for *S. epidermidis*, *p* = 0.32 for *S*. Enteritidis). Mean ± SD for *n* = 3 is shown. **c** Optoplots showing the fluorescence intensity (RFU) of HS-167 in bTSB cultures of *S. aureus* (clear circles), *S. epidermidis* (gray circles) and *S.* Enteritidis (black squares). *n* = 3. **d** Slopes extracted from the optoplots presented in **c**. Data were analysed by ANOVA (*F* = 68.44, *p* < 0.0001) and Tukey´s post-hoc test (*S. aureus* vs. *S.* Enteritidis *p* < 0.0001; *S. aureus* vs. *S. epidermidis* *p* = 0.0032*; S. epidermidis* vs. *S.* Enteritidis *p* = 0.0022). Lines show mean, error bars show ± SD.

To enable high-throughput screening, we next developed a workflow consisting of an *Experimental procedure* followed by *Automated data analysis* (Fig. 2a). The experimental part covers the preparation of bacterial cultures and optotracing. Bacteria from overnight cultures in TSB were diluted in bTSB containing HS-167, aliquots were dispensed in 96-well plates, which were incubated for ca 12 h at 37 °C in a plate reader. Every 15 min, we measured A_600_ and emitted fluorescence from HS-167 (Ex. 507 nm and Em. 625 nm). These data were fed into the *Automated data analysis*, performed using Python programming language. To determine the exponential phase, we smoothed the growth curves using Savitzky–Golay digital filter and identified the parts exhibiting the fastest rate of increase. We defined the exponential phase to be within the top 20% of the gradient of A_600_. Extraction of A_600_ and fluorescence values from the selected time frame generated data that were processed in two parallel tracks: (i) to calculate the generation time of each culture, and (ii) to generate optoplots for each culture, from which the slope, calculated by linear regression of the smoothed fluorescence intensity versus A_600_, was used for comparison between samples. We tested this workflow by growing cultures of *S. aureus* and *S. epidermidis* in bTSB ± HS-167, using cultures of *S.* Enteritidis as a negative control (Fig. S3). Automated data analysis revealed that HS-167 did not alter the growth rate in any of the strains (Fig. 2b). By visualizing the fluorescence in relation to A_600_ for each culture, the optoplot revealed a negative slope (−195.1 ± 23.6 RFU/AU) for *S*. Enteritidis (Fig. 2c, d). This may originate from an increased bacterial scattering of the weak fluorescence from unbound HS-167, or from photobleaching of the unbound dye under conditions used. In contrast, both *Staphylococcal* species showed positive slopes. *S. aureus* presented a slope of 2757 ± 533 (RFU/AU), which was 2× higher than that of *S. epidermidis* (1331 ± 40 RFU/AU). Importantly, results from these real-time recordings in liquid cultures match those from optotracing of serial dilutions of bacteria in PBS (Fig. 1h). By combining a minimum of wet-lab time and solid *Automated data analysis*, we have established an optotracing-based workflow for high-throughput screening of bacterial cultures in real-time.

### Optotracer binds cell wall components of *Staphylococci*

**Fig. 3:**
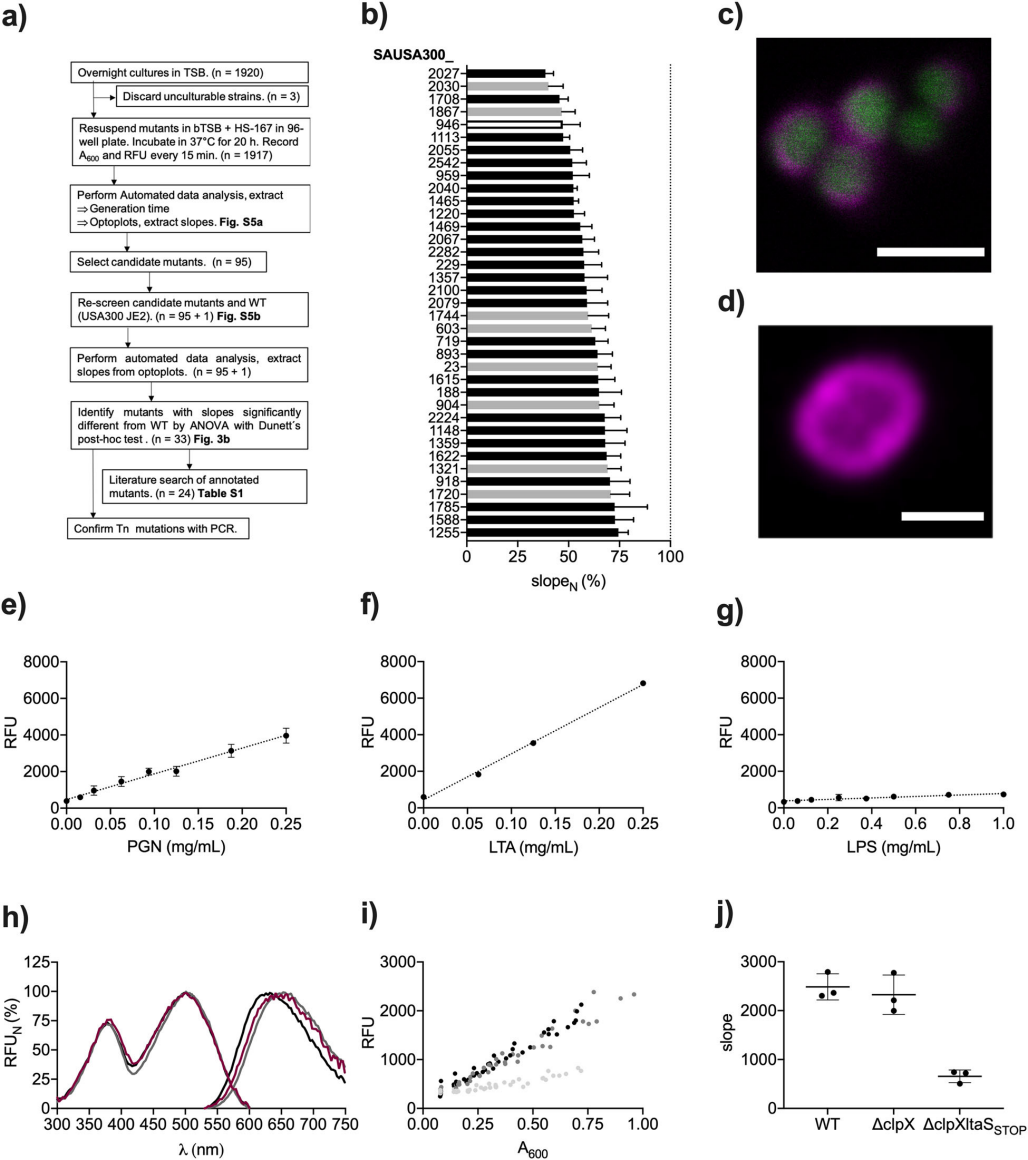
Identification of binding targets for HS-167 on *Staphylococci*. **a** Flowchart of the screening process of Tn mutants with altered HS-167 binding. **b** List of 37 mutants selected from the Tn library whose normalized slope (slope_N_) differs significantly from the WT, as determined by one-sample *t*-tests (*p* ≤ 0.0001). Strains with mutations in annotated sequenced (black), nonsequenced (white), and nonannotated (gray) genes are shown. Mean from *n* = 4 + SD is shown. **c** Confocal microscopy of HS-167 bound to GFP-expressing *S aureus* 8325-4. Magenta = fluorescence of HS-167 bound to the cell wall, green = GFP. Scale bar = 2 µm. **d** Airyscan image of HS-167 bound to *S. epidermidis* (magenta). Scale bar = 1 µm. **e**–**g** Fluorescence intensity of HS-167 (10 µM in PBS) at Ex. 507 nm and Em. 625 nm, at different concentrations of **e** PGN from *S. aureus* (*n* = 3), **f** LTA from *S. aureus* (*n* = 2), and **g** LPS from *S.* Enteritidis (*n* = 3). Filled circles show mean, error bars show ± SD. **h** Normalized specplots of HS-167 bound to *S. aureus* cells (magenta, *n* = 3), PGN (black, *n* = 3), and LTA (dark gray, *n* = 2). Lines show mean values. **i** Fluorescence intensity (RFU) from HS-167, recorded at Ex. 507 nm and Em. 625 nm, at different cell densities (A_600_) of *S. aureus* WT (black), *S. aureus* Δ*clpX* (dark gray) and *S. aureus* Δ*clpXltaS*_*STOP*_ (light gray) in PBS + HS-167 (10 µM). All experimental points are shown from *n* = 3. **j** Slopes extracted from **i**. Slopes were compared by ANOVA (*F* = 37.13, *p* = 0.0004) and Tukey´s post-hoc test (*S. aureus* WT vs. *S. aureus* Δ*clpXltaS*_*STOP*_ *p* = 0.0006; *S. aureus* WT vs. *S. aureus* Δ*clpX* *p* = 0.78; *S. aureus* Δ*clpX* vs. *S. aureus* Δ*clpXltaS*_*STOP*_ *p* = 0.001). Lines show mean, error bars show ± SD.

To identify the binding target of HS-167 on *S. aureus*, we applied the optotracing high-throughput workflow to screen a *bursa aurealis* Tn library with defined mutations in nonessential genes. As this is based on *S. aureus* strain USA300 JE2, we first ensured that this strain showed the expected growth characteristics in bTSB ± HS-167 (Fig. S4a–d). As indicated in the screening overview (Figs. 3a), 3 of the 1920 mutants were unculturable and had to be discarded, the remaining 1917 mutants were prepared for cultivation in bTSB + HS-167 in 96-well plates. Mutants were incubated for 20 h to allow all strains, irrespective of generation time, to pass the exponential phase. As A_600_ and emitted fluorescence were fed into the *Automated data analysis*, all mutants showed positive slopes in the optoplots (Fig. S5a). Since HS-167 was able to bind all strains with mutations in nonessential genes, we suggest that the binding target is a product of an essential gene. Based on the optoplot analysis, we selected 95 candidate mutants representing those with the highest and lowest slopes of the library (Fig. S5a). We undertook a repeated screen of these mutants, including the parental wild type (WT) strain, which enabled us to normalize the slopes of mutants in each screen to that of the WT (Fig. S5b). To distinguish mutants with slopes different from WT, we performed a one-sample *t*-test (*α* = 0.0001) for each mutant, which identified 37 mutants, all presenting lower slopes than WT (Fig. 3b). When analyzing the functions of the disrupted genes, we focused on the 29 mutants with annotated genes. Of those, 25 clustered into one or more group(s) linked to cell envelope (18 mutants), amino acids (four mutants), central metabolism (two mutants), and global regulators (three mutants) (Table S2). Thus, a majority of genes were directly or indirectly involved with the cell wall. Mutants with the lowest slope, representing cells with ≤50% binding of HS-167 relative to the WT, were Δ*alr* (SAUSA300_2027), Δ*rot* (SAUSA300_1708), Δ*pknB* (SAUSA300_1113), Δ*menD* (SAUSA300_0946) and Δ*murA* (SAUSA300_2055) (Fig. 3b). The gene *murA* exists in two copies, *murA* and *murA2*. The one causing reduced binding of HS-167 was *murA*, which is known to reduce the peptidoglycan (PGN) content of cell walls^18^. In contrast, no such effects are known for Δ*murA2*, which explains why this mutant (SAUSA300_2078) did not appear among the group of 95 candidate mutants. A low slope was also observed for *pknB*, which acts downstream of *murA* controlling the expression of *murC*, *murD*, and *murF*. The *pknB* encoded serine–tyrosine kinase is important for cell wall homeostasis, cell division, autolysis, and susceptibility to β-lactams. The gene *menD* encodes 2-succinyl-5-enolpyruvyl-6-hydroxy-3-cyclohexene-1-carboxylate synthase involved in menaquinone biosynthesis. Deletion of this gene decreases the membrane potential, rendering bacteria more resistant to aminoglycosides, and small colony variants often show mutations in *menD*^19^. The stationary phase transcription factor Rot is reported to regulate the expression of virulence genes and biofilm formation^20,21^. The mutant Δ*alr* showed the lowest slope of all mutants. The encoded racemase, which is constitutively expressed in *S. aureus*, converts L-ala to D-ala for incorporation into PGN, wall teichoic acids (WTA), and lipo-teichoic acids (LTA). In contrast, the inducible homolog *alr2* (Δ*alr2*, SAUSA300_1292) did not appear among the 95 candidate mutants. Taken together, as our exhaustive analysis of almost 2000 genes narrows in on a subset predominantly related to the cell envelope, we find this essential structure a plausible binding target for HS-167. To test this hypothesis, we examined the fluorescence pattern of GFP-expressing *S. aureus* stained with HS-167. In confocal microscopy, HS-167 fluorescence was localized to the cell envelope, surrounding the intracellular GFP signal (Fig. 3c). The pattern was verified with super-resolution microscopy, showing HS-167 staining of the cell wall including the division septum of *S. epidermidis* (Fig. 3d). Thus, our genetic and microscopy approaches coherently identify the cell wall as a binding target for HS-167.

The major constituent of gram-positive cell walls, PGN, consists of alternating residues of β-(1,4) linked N-acetylglucosamine and N-acetylmuramic acid. As HS-167 was added to serially diluted PGN, a linear increase of emitted fluorescence was observed, indicative of binding (Fig. 3e). LTA is also a common wall-associated constituent. Although considered anionic, this polymer is decorated with positively charged D-ala^22^. As for PGN, a linear correlation was observed between the concentration of LTA and fluorescence intensity from HS-167 (Fig. 3f). Lipopolysaccharide (LPS) from gram-negative bacteria is hydrophobic and negatively charged, similar to LTA. However, LPS is also highly amphiphilic, promoting arrangements in defined orders in aqueous solutions^23^. When performing optotracing of LPS from *S.* Enteritidis, HS-167 showed a negligible signal (Fig. 3g). Thus, HS-167 is unable to bind LPS, which explains the lack of signal from HS-167 mixed with *Salmonella* cells.

To better understand the interaction between HS-167 and gram-positive cell wall components, we compared the excitation spectra in normalized spec-plots of HS-167 bound to PGN and LTA, to that of HS-167 binding to *S. aureus*. An overlap was observed for all three, implying that HS-167 adopts a similar conformation when binding to all three targets (Fig. 3h). To analyze the role of LTA for HS-167 binding to bacterial cells, we complemented the Tn library by the strain *S. aureus* Δ*clpX ltaS*_STOP_. This strain produces viable LTA-deficient cells as its truncated LTA synthase gene is accompanied by a deletion of the *clpX* chaperone^24^. For comparison, we included *S. aureus* WT and *S. aureus* Δ*clpX* when recording fluorescence and A_600_ from serial dilutions of each strain in the presence of HS-167 (Fig. 3i). Optoplot analysis showed that the slope of the LTA-deficient strain was reduced by ca 74% compared to the slopes of WT and *S. aureus* Δ*clpX* (Fig. 3j). While the greatly attenuated slope identifies LTA as a major binding target for HS-167 on bacterial cells, the remaining signal indicates that PGN and possibly WTA also contribute to the binding of HS-167 to *Staphylococci*.

### Optotracing discriminates *E. faecalis* from *S. aureus*

**Fig. 4:**
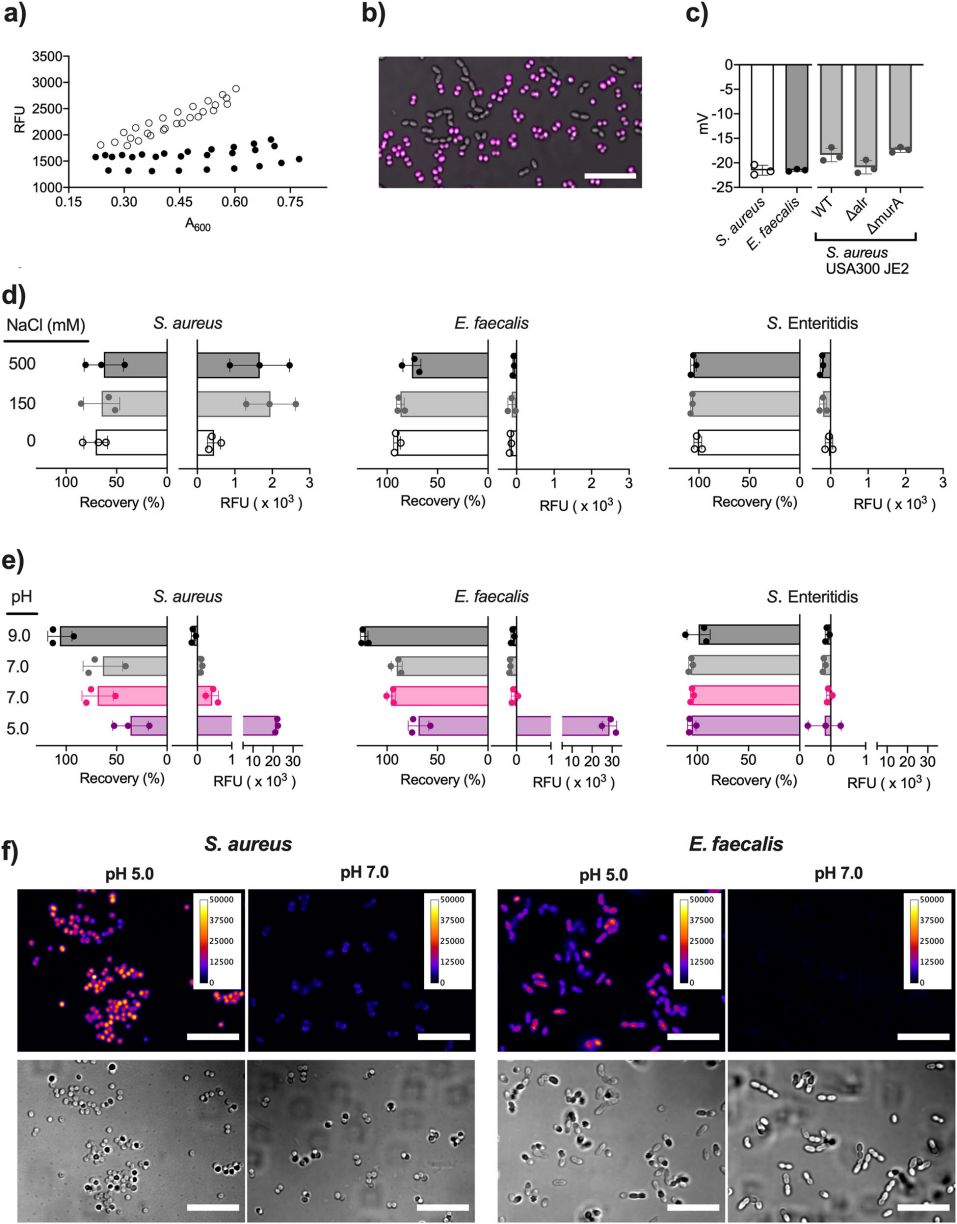
Binding of HS-167 to bacteria is governed by hydrophobic interactions. **a** Optoplots of *E. faecalis* (black) grown in bTSB with added HS-167 (*n* = 3). Corresponding optoplots for *S. aureus* (adapted from Fig. 2c) are shown (white) for comparison. **b** Merged transmitted light and pseudocoloured confocal images of HS-167 (magenta) in a mixed sample of *S. aureus* and *E. faecalis*. Scale bar = 10 µm. **c** Zeta potential of *S. aureus* and *E. faecalis* in 10 mM PB, the Tn mutants with reduced binding of HS-167, and the WT USA300 JE2. Bars = mean ± SD from *n* = 3, circles = the individual data points. *S. aureus* and *E. faecalis* were compared by a *t*-test (df = 4, *p* > 0.99). *S. aureus* USA300 JE2 WT and the two mutants were compared by ANOVA (*F* = 7.481, *p* = 0.0235) and Dunnett’s post-hoc test (WT vs. Δ*alr* *p* = 0.0657; WT vs. Δ*murA* *p* = 0.4756). **d**, **e** Bacterial adhesion to hexadecane (Recovery, left graph) and the fluorescence intensity (RFU, right graph) from HS-167 added to *S. aureus*, *E. faecalis* and *S*. Enteritidis in **d** 20 mM sodium phosphate/citric acid buffer pH 7.0 with 0 mM (white), 150 mM (gray) and 500 mM (black) NaCl, and **e** in 20 mM sodium phosphate/citric acid buffer pH 5.0 (purple), pH 7.0 (pink) and 20 mM Tris/hydrochloric acid buffer pH 7.0 (gray), 9.0 (black) Bars = mean ± SD from *n* = 3, circles = the individual data points. **f** Images from confocal (top) and transmitted light (bottom) microscopy of *S. aureus* and *E. faecalis* in sodium phosphate/citric acid buffer with 150 mM NaCl at pH 5.0 and pH 7.0. Calibration bars show fluorescence intensity. Scale bar = 10 µM.

The ubiquitous presence of PGN and LTA in cell walls of gram-positive bacterial species led us to investigate if HS-167 can be used to stain other gram-positive bacteria. Since *Enterococcus faecalis* (*E. faecalis)* express similar LTA as *S. aureus* (Type I)^25,26^, we performed real-time optotracing of this strain with HS-167. Automated data analysis showed no linear correlation between fluorescence intensity and A_600_, suggesting an inability of HS-167 to bind this species (Fig. S6a and Fig. 4a). To confirm this, we resuspended cells in PBS + HS-167, and performed a spectral analysis in a plate reader. Spec-plot analysis showed similarly low fluorescence intensity from *E. faecalis* mixed with HS-167 as from the PBS + HS-167 control (Fig. S6b). Lack of HS-167 binding to *E. faecalis* was further verified by confocal microscopy of a mixed species sample, wherein diplococci and short chains of *E. faecalis* remained unstained, while coccoid *S. aureus* showed bright fluorescence from bound HS-167 (Fig. 4b).

Differential binding of HS-167 to *S. aureus* and *E. faecalis* may be explained by different physico-chemical properties of the respective cell wall, such as surface charge and hydrophobicity. To analyze the net surface charge of *S. aureus* and *E. faecalis*, we measured the zeta potential. No difference was observed as both strains showed a mean zeta potential of −21.5 mV (Fig. 4c). To confirm that HS-167 binding occurs independent of the zeta potential, we analyzed two of the Tn mutants with attenuated HS-167 binding (Δ*alr*, Δ*murA*). Both strains showed similar zeta potential as the WT, suggesting that differential HS-167 staining cannot be ascribed to differences in the net surface charge of cells.

We next analyzed the role of hydrophobic interactions using the bacterial adhesion to hydrocarbons assay (BATH), which provides a relative measure of hydrophobicity^27^. Hydrophobic bacteria associate with hexadecane, while hydrophilic bacteria remain in the aqueous phase from where they can be recovered. We evaluated the hydrophobicity of *S. aureus* and *E. faecalis*, using *S.* Enteritidis as control. In neutral buffer (0 mM NaCl), we found *S. aureus* (70.7 ± 11.7%) to be most hydrophobic, *E. faecalis* (90.2 ± 3.3%) as intermediate, and *S.* Enteritidis (101 ± 3.8%) as most hydrophilic (Fig. 4d). The difference between *S. aureus* and *E. faecalis* suggest a possible role for hydrophobicity in the differential HS-167 staining of these species. To further enhance the hydrophobic interactions, we increased NaCl to 150 and 500 mM and measured bacterial hydrophobicity as well as binding of HS-167 to the cells. BATH analysis showed that higher osmolarity barely affected *S. aureus* (<10% reduction in recovery at both concentrations). *E. faecalis* showed similarly small reduction (4% and 15%, respectively), whereas neither concentration influenced the hydrophobicity of *S.* Enteritidis. Fluorescence intensity recordings at high salt still showed selective binding of HS-167 to *S. aureus*. Compared to no salt, the signal intensities were increased by ca 340%. This is more than ten times higher than the salt effect on the fluorescence intensity of unbound HS-167 (26%) (Fig. S7a). High osmolarity thus causes a relatively small increase of the intrinsic fluorescence of the optotracer, while at the same time promoting binding of the optotracer to the target cell. Collectively, the increased proportion of bound vs. unbound optotracers generates a higher signal-to-noise ratio and presumably a lower detection limit. Moreover, as the increased salt concentration appeared to strengthen rather than attentuate the binding of HS-167, we suggest that the binding is less likely to occur through electrostatic interactions.

The minor effect of added salt on bacterial hydrophobicity prompted us to alter the pH as a means to modify hydrophobic interactions. In BATH assays, *S. aureus* showed low recovery (36 ± 17.6%) at pH 5.0, which increased to ca 65% at pH 7.0, reaching ca 100% at pH 9.0 (Fig. 4e). A similar trend was observed for *E. faecalis*, whose low recovery at pH 5.0 (68.9 ± 10.2%) increased to ca 93% at pH 7.0, reaching ca 100% at pH 9.0. As expected, *S.* Enteritidis showed ca 100% recovery regardless of pH, supporting the highly hydrophilic nature of these gram-negative bacteria. By recording the fluorescence intensity, we observed strong binding of HS-167 to *S. aureus* at pH 5.0 (21,869 ± 693.2 RFU), which was significantly lowered at pH 7.0, and abolished at pH 9.0. Very high fluorescence intensity was also observed for HS-167 mixed with *E. faecalis* at pH 5.0 (28,707 ± 3548.4 RFU), suggesting efficient binding to this species at acidic pH. The signal disappeared at pH 7.0 and pH 9.0. For *S.* Enteritidis, no signal was observed irrespective of the buffer used. Collectively, this defines hydrophobic interactions as the primary binding mode of HS-167 to *S. aureus* and *E. faecalis*. At pH 5, protonation of bacterial surface components reduces the electrostatic repulsion, leading to increased hydrophobicity and low BATH recovery. This increases the affinity of HS-167 for its target, generating fluorescence signals from HS-167 interacting with *S. aureus* as well as *E. faecalis*. In contrast, cells are highly negatively charged at pH 9, when the hydrophilic nature of cells leads to full BATH recovery, and electrostatic repulsion hinders binding of HS-167. To investigate if these findings translate to fluorescence microscopy, we imaged *S. aureus* and *E. faecalis* mixed with HS-167 in buffers at pH 5.0 and 7.0. The intensity of emitted fluorescence from HS-167 bound to *S. aureus* was much higher at pH 5.0 than at pH 7.0 (Fig. 4f). Similarly, an intense signal from HS-167 bound to *E. faecalis* was observed at pH 5.0, and this signal was fully eliminated at pH 7.0, as expected from the spectroscopic recordings. Collectively, this shows that HS-167 binds bacterial cells predominantly via hydrophobic interactions.

## Discussion

We demonstrate optotracing as a new method for rapid detection of *S. aureus* and *S. epidermidis*. Binding of optotracers to the *Staphylococcal* cell wall serves as an on-switch of fluorescence detectable by microscopy and spectroscopy. This makes optotracing a highly versatile method, equally applicable for in vitro staining of bacterial cells, as for real-time analysis of live bacterial cultures. Unique to optotracers is the inducible fluorescent signal, which remains off until the biomolecular binding target appears. This switch enables real-time quantification of *Staphylococci* in liquid cultures, offering a fluorescence-based alternative to absorbance recordings. Detection and quantification of *Staphylococci* is also achievable when mixed with gram-negative bacteria, since the optotracer only binds to the gram-positive cell wall. Binding selectivity can be tuned to distinguish species within the gram-positive group, as shown for *S. aureus* and *E. faecalis*. Optotracers bind selectively to *Staphylococci* at neutral pH, while reporting of both species is achieved by increasing the hydrophobic interactions using acidification. By integrating algorithm-based analytics, we translate optotracing into a rapid and automated high-throughput platform for bacterial detection and identification.

While optotracing was initially developed for detection and identification of ECM constituents of biofilm-forming *S.* Enteritidis^14^, we here evolved this technique for detection of polymeric structures in the cell wall of *Staphylococci*. Real-time recordings in live cultures can be achieved since the optotracer does not affect bacterial growth, and its fluorescence is only induced upon binding to bacteria. While AIE luminogens also possess an on-switch capability, the mechanism differs^6^, and they are primarily used in antibacterial applications^6,28^. The fluorescence intensity of other types of fluorophores coupled to antibodies or other recognition molecules is typically unaffected by the binding event and can therefore not be used for in situ quantification. HS-167 is a valuable tool for fluorescence microscopy, as the wide Stokes shift allows combined staining using HS-167 and conventional fluorophores without spectral overlapping, as was shown for intracellular GFP and cell wall-associated HS-167. Moreover, HS-167 showed much better photostability compared to GFP. The small-sized optotracers are also useful for super-resolution imaging, as previously shown for amyloid fibrils imaged by binding activated localization microscopy^29^. We demonstrate the use of optotracers in super-resolution imaging of bacterial cells by AiryScan, confirming localization of HS-167 to the cell wall of *S. epidermidis*. Despite the important role of the cell envelope, relatively few methods exist to image this complex and dynamically changing structure. Commonly used cell wall targeting antibiotics coupled to fluorophores are of limited use as they inhibit bacterial growth^30^. Fluorophore-coupled wheat germ agglutinin is compatible with bacterial growth and works well on gram-positive bacteria but cannot be used to distinguish between species. Fluorescent D-amino acids (FDAA) have furthered our understanding of the cell wall, but kinetic experiments are hindered as the required high concentration of FDAA introduces a washing step prior to imaging. Optotracing supplements these existing techniques, especially for studies of live bacteria and for kinetic studies.

Previous studies have mainly analysed the spectral shifts when demonstrating binding of optotracers to target molecules^14,16,31,32^. As we here utilize a new class of optotracers with an on-switch of fluorescence, we used fluorescence intensity as the primary determinant of the binding event. Simultaneous recording of absorbance and fluorescence is easily achieved in a multimode microplate reader, a format that also facilitates high-throughput experimentation with minimal wet-lab time.

In our search for the HS-167 binding target on *Staphylococci*, multiple techniques were applied which collectively pointed at the cell wall. In our fully unbiased screen of 1917 Tn mutants, HS-167 was found to bind all strains, implying that the binding target is a product of an essential gene(s). Several of the mutants showed, however, attenuated fluorescence (i.e., less binding of HS-167) and >60% of those were related to the cell envelope. Previous studies reported 66 essential genes annotated for a role in the cell envelope of *S. aureus* SH1000^33^, of which eight were reported as nonessential in USA300 JE2^34^. We identified five of the nonessential eight genes to exhibit reduced binding of HS-167. Collective results from the Tn screen pointed towards the cell envelope as a plausible target for HS-167 binding. Use of isolated compounds showed HS-167 binding to PGN and LTA, but not to LPS. This was corroborated by the reduction of HS-167 binding to the mutant *S. aureus* 8325-4 Δ*clpX ltaS*_STOP_, known for its altered PGN structure and lack of LTA^24^.

Super-resolution microscopy revealed an even distribution of HS-167 fluorescence throughout the cell envelope, strengthening our hypothesis of HS-167 binding to a structural component of the cell wall rather than a surface-bound molecule. The size of the pseudo-tessera in the PGN of *S. aureus* is reported to be 23 Å^35^. This is significantly smaller than in *B. subtilis*, whose tessera was estimated to circa 50 Å^35,36^, and we therefore propose that the small, <1 kDa, size of HS-167 is critical for the molecule to gain access to the interior of the PGN layer. Furthermore, we suggest that the negative charge of the molecule provides selective affinity to *S. aureus* at neutral pH, where the electrostatic repulsion is too prominent for HS-167 to bind other gram-positive species, such as *E. faecalis*. While a wider screen, including numerous strains of gram-positive and gram-negative species needs to be performed to gain a better insight into the mechanism of binding, we view the pH dependent binding as an advantage for future diagnostic applications. Moreover, current restrictions imposed by the pH-sensitive binding, e.g., for in situ recordings within dynamically changing microenvironments, can likely be solved by further chemical modifications of the optotracer.

Optotracing is a potent technology under development, and insight from this work will aid in structure optimization of optotracers. By controlling the length, molecular composition, and charge distribution along the conjugated backbone, molecules with tailored binding specificity and improved environmental stability can be developed. We envisage that optotracing will develop into a technologically advanced, yet simple to use, fluorescence microscopy technique for live imaging of bacteria in sessile and planktonic life styles, and as an automated method for identification of different bacterial species.

## Methods

### Chemicals

Chemicals, including peptidoglycan (PGN), lipoteichoic acid (LTA), lipopolysaccharide (LPS), were obtained from Sigma-Aldrich (Sweden) unless otherwise stated. Phosphate buffer saline (PBS) pH 7.4 (Medicago, Sweden) was prepared in ultrapure water and autoclaved before use. Optotracers were synthesized as described^10^, kept at 1.5–1.65 mM in ultrapure water and used at 5 µM unless stated differently.

### Bacterial strains, plasmids, and growth media

Bacterial strains, listed in Table S1, were maintained as stocks in −80 °C. The plasmid pSGFPS1^37^ was used to create GFP expressing *S. aureus* 8325-4, using trimethoprim (25 µg/mL) supplemented cultures when needed. Tryptic Soy Broth (TSB) and Brain Heart Infusion broth (BHI) were prepared according to suppliers’ instruction (Sigma-Aldrich, Sweden). Bacterial cultures were incubated at 37 °C under shaking (160 rpm) conditions unless otherwise stated, using a single colony from strains grown overnight on Tryptic Soy Agar as inoculum of 5 mL medium. TSB was used for all *S. aureus* strains*, S. epidermidis* and *S.* Enteritidis. *E. faecalis* was grown statically in BHI when preparing for fluorescence spectroscopy, microscopy and zeta potential measurements. Shaking TSB cultures were used for real-time optotracing.

To establish and perform the high-throughput workflow, buffered TSB (bTSB, pH ≈ 7.5) was prepared by adding 10 g/L dibasic potassium phosphate and 1 g/L monobasic potassium phosphate to TSB, which then was filter-sterilized (0.2 μm, Sarstedt, Germany). bTSB was used for overnight cultures of *S. aureus* 8325-4 WT, Δ*clpX* and Δ*clpXltaS*_STOP_ (static conditions), and the real-time optotracing assay.

Growth rate and pH of *S. aureus* 8325-4 (Fig. S2b–d) and *S. aureus* USA300 JE2 (Fig. S4a–c) were determined in 150 mL cultures of TSB or bTSB, prepared by 1:100 dilution of overnight cultures. The number of biological replicates is stated in the respective figure legend (n number). Every 30–40 min, we collected 6 mL from each culture and measured the absorbance at 600 nm (A_600_), determined CFU counts and measured the pH of the supernatant. The generation time of *S. aureus* 8325-4, *S. epidermidis*, *S.* Enteritidis (Fig. 2b) and *S. aureus* USA300 JE2 (Fig. S4d) in 96-well plates was determined in 200 μL cultures, prepared by 1:1000 dilution of overnight cultures, incubated at 37 °C in a Synergy™ MX plate reader (BioTek, USA) with A_600_ recorded every 15 min. The exponential growth phase, determined to be the top 20% (for 12 h growth curves) or top 10% (for 20 h growth curves) of the gradient of the smoothed A_600_ was used to calculate the generation time.

### Optotracing by fluorescence spectroscopy

To prepare samples for endpoint fluorescence spectroscopy, overnight cultures were washed twice and resuspended in PBS or other buffers as indicated. Following the addition of optotracers to each sample, 200 µL was transferred to flat bottom transparent 96-well plates (Sarstedt, Germany). Technical triplicates were performed except for experiments with PGN, LTA, LPS, and *S. aureus* 8325-4 WT, Δ*clpX*, and Δ*clpX*ltaS_STOP_, which were assayed in technical duplicates. Technical multiplicates were averaged for each biological replicate. The numbers of biological replicates for each experiment are stated in the respective figure legends (*n* number). Excitation and emission spectra were recorded in a Synergy™ MX plate reader (BioTek, USA) using 9 nm margin and ≤3 nm step size. The following settings were used: HS-84: excitation at 300–500 nm, emission collected at 535 nm; emission at 450–750 nm using 425 nm excitation, at 80% sensitivity. HS-163: excitation at 300–470 nm, emission collected at 492 nm; emission at 420–750 nm using 395 nm excitation, at 80% sensitivity. HS-167: excitation at 300–600 nm, emission collected at 625 nm; emission at 530–750 nm using 507 nm excitation. HS-167 was also used to record fluorescence endpoint values (507 nm excitation, 625 nm emission). A margin of 20 nm was used for single measurements, while 9 nm was used in the high-throughput, real-time optotracing protocol. A margin of 9 nm was used for single measurements in experiments with PGN, LTA, LPS, and *S. aureus* 8325-4 WT, Δ*clpX* and Δ*clpXltaS*_*STOP*_.

### Fluorescence microscopy

To prepare samples for fluorescence microscopy, overnight cultures were washed twice and resuspended in indicated buffers. For microscopy in sodium phosphate/citric acid buffers with different pH, exponential cultures of *E. faecalis* were used to avoid artefacts from the entry of HS-167 in dead *E. faecalis* cells. After addition of the optotracer, samples were incubated approx. 45 min at room temperature. Aliquots of 5–7 µL were withdrawn and placed directly onto a microscope slide, and the added glass cover slip was sealed with nail polish. Confocal microscopy was performed at Biomedicum Imaging Core facility (Karolinska Institutet, Sweden). *S. aureus, S.* Enteritidis, *E. coli, S. epidermidis*, and *E. faecalis* were imaged in PBS with added optotracer on an Olympus FV1000 with 60× water objective. The 405 nm excitation laser was used for HS-84 and HS-163, with emission collected at 490–590 nm (HS-84) and 490–540 nm (HS-163). For HS-167, we used 473 nm excitation laser, collecting emission at 575–675 nm. GFP-expressing *S. aureus* in PBS was imaged on a Zeiss LSM800 using 100× oil immersion objective. The same set-up was used for *S. aureus* and *E. faecalis* in sodium phosphate/citric acid buffers. Imaging of GFP-expressing *S. aureus* in PBS + HS-167 was performed using 488 nm excitation laser for GFP and 561 nm laser for HS-167. A short pass filter below 535 nm was used to collect GFP emission and a long pass filter above 625 nm for HS-167 emission. To visualize the relative difference in fluorescence intensity from HS-167 bound to *S. aureus* and *E. faecalis*, we used sodium phosphate/citric acid buffer (pH 5.0 and 7.0) with 150 mM NaCl. We used the 488 nm excitation laser and a long pass filter above 580 nm for detection of emitted fluorescence. Super-resolution microscopy of *S. epidermidis* stained with HS-167 was performed at the Advanced Light Microscopy facility at Science for Life Laboratory (Stockholm). Using a Zeiss LSM880 equipped with an Airyscan detector and 63× oil immersion objective, imaging was performed in sodium phosphate/citric acid buffer, pH 5.0, 150 mM NaCl. The 488 nm excitation laser was used, with emission collected at 495–550 nm and above 570 nm. Deconvolution was performed using Zen software from Zeiss microscopy. FIJI^38^ imaging software was used to process all images. Pseudocolors were arbitrarily assigned to facilitate differentiation of the optotracers. Representative images from biological replicates of *n* ≥ 3 are shown for all experiments except for superresolution imaging by AiryScan (*n* = 1).

### Real-time optotracing by 96-well assay with automated data analysis

From overnight cultures diluted 1:1000 in bTSB ± HS-167, samples (200 µL) were pipetted in flat bottom transparent 96-well plates (Sarstedt, Germany). Technical triplicates were used for *S. aureus*, *S. epidermidis*, *S.* Enteritidis, and *E. faecalis*, while strains from the Tn library were assayed in singlets. The initial screen of 1920 transposon mutants was performed in a single replicate, all other experiments were performed in biological multiplicates, see respective figure legends for *n* numbers. Plates were incubated in Synergy™ Mx Microplate Reader (BioTek, USA) at 37 °C without shaking, with A_600_ and fluorescence measured every 15 min. Automated data analysis and visualization was performed with Python programming language using SciPy^39^, NumPy^40^, and Matplotlib^41^ packages. Data (A_600_, RFU) obtained from the high-throughput, real-time optotracing experiments was filtered using a Savitzky-Golay filter with the polynomial order of 3. The length of the data window was 13 for *S. aureus*, *S. epidermidis*, *S.* Enteritidis, and *E. faecalis*, and 27 for the Tn library. To define the exponential phase, we calculated the gradient (i.e., direction and rate of fastest increase) of the filtered A_600_ data within the time frame of the top 20% values of the gradient for *S. aureus*, *S. epidermidis*, *S.* Enteritidis, and *E. faecalis*, and the top 10% for the Tn library. Generation times were calculated within the exponential phase. Optoplots were generated by plotting fluorescence values (RFU) versus A_600_ (AU) during the exponential phase of growth. Linear regression was performed and the slopes of the optoplots were extracted.

### Transposon mutant library

The Nebraska Transposon Mutant Library^34^ includes 1920 strains, each with a mutation in a nonessential gene. Mutants in the original 384-well plates were transferred to glycerol stocks in 96-well plates, which we stored at −80 °C. To grow overnight cultures, we used a replicator to inoculate TSB-containing (200 μL) wells in 96-well plates, incubated the plates at 37 °C for 18 h in an Infinite M1000 Pro microplate reader (Tecan, Männedorf, Switzerland) and monitored growth by measuring A_600_ every15 min. From all wells of the overnight cultures, we diluted samples 1:1000 in bTSB + HS-167 and proceeded with the screening procedure (described in “Real-time optotracing by 96-well assay with Automated data analysis”) using 20 h incubation. Four candidate mutants were selected as the mutants with the highest (2) and lowest (2) fluorescence from each plate, generating 80 mutants in total. Additionally, we selected ten mutants with lowest and five with highest slopes based on the pooled slopes from all plates. Glycerol stocks of each the 95 selected mutants and of the parental strain USA300 JE2 were stored at −80 °C. These 96 strains were rescreened four times and relative slopes of the mutants compared to the WT were extracted for every screen.

We sequenced the annotated mutant strains with slopes significantly different from the WT to confirm the Tn mutation. Briefly, we isolated genomic DNA using GenElute™ Bacterial Genomic DNA Kit. Restriction and ligation of DNA samples was performed using AciI restriction enzyme and T4 DNA ligase as described^1^. DNA fragments around the *bursa aurealis* transposon were amplified with polymerase chain reaction (PCR) reaction using the Buster and Martn-ermR primer set. PCR reaction included 30 cycles, annealing temperature of 64.3 °C, 3 min extension time. Gel electrophoresis (1% agarose) confirmed successful PCR reaction in all mutant strains, except for SAUSA300_0946. PCR products were purified using GFX^™^ PCR DNA and Gel Band Purification Kit (GE Healthcare, UK) and ExoSAP-IT^™^ PCR Product Cleanup Reagent (ThermoFisher, Sweden). Nucleotide sequences of DNA spanning the transposon were determined using Buster primer (Eurofins, Germany).

### Zeta potential measurements

Zeta potential was recorded with Zetasizer Nano (Malvern, UK), using DTS1070 cuvettes (Malvern Panalytical, UK). Bacterial overnight cultures were washed twice in 10 mM sodium phosphate/citric acid buffer pH 7.0 and diluted to OD_600_ ~ 0.15. Water was chosen as a dispersant in the software setup, Smulchowski approximation was selected, *F*(ka) value = 1.5. The temperature during recordings was 25 °C, 120 s equilibration time. Attenuation (11–0) and run selection (10–100) was automatic, the voltage was set to 25 V. Measurements were performed in biological triplicates with each sample analyzed in four subsequent measurements.

### BATH assay and fluorescence recordings

Sodium phosphate/citric acid buffers (20 mM, pH 3.0, 5.0, 7.0) were prepared by mixing 0.1 M citric acid and 0.1 M dibasic sodium phosphate (Merck, Germany) to the desired pH. Trizma/hydrochloric acid buffers (20 mM, pH 7.0, 9.0) were prepared by equilibrating 0.1 M Trizma buffer with concentrated hydrochloric acid. The salt concentrations were adjusted to 150 and 500 mM using 2.5 M NaCl. All buffers were autoclaved before use. BATH assay was performed as described^27^. Briefly, overnight cultures of *S. aureus, E. faecalis*, and *S.* Enteritidis were washed twice in buffers with defined salt concentration and pH and resuspended in the same buffer to A_600_ ≈ 0.5. From the resuspended culture, 3 mL was transferred to an acid-washed glass tube, 0.5 mL hexadecane was added, and the mix was vortexed for 90 s. Tubes were left to stand for 20 min to allow phase separation. Using a Pasteur pipette, we recovered the aqueous phase and measured A_600_. Recovery was calculated as the ratio between A_600_ of the recovered aqueous phase and that of the culture before mixing with hexadecane. To enable correlation of results from the BATH assay and HS-167 binding to cells, we added HS-167 to resuspensions of bacterial cultures in the designated buffers (A_600_ ≈ 0.5) and recorded the fluorescence intensity of technical triplicates in a Synergy™ MX plate reader (BioTek, USA). The experiment was performed in biological triplicates, as stated in the figure legend.

### Software, data analysis, and statistics

Chemicalize was used for prediction of pH dependent charge distribution (Chemicalize, January 2019, https://chemicalize.com/) developed by ChemAxon (https://www.chemaxon.com). Automated data analysis was performed as described in “Real-time optotracing by 96-well assay with Automated data analysis”). Linear regression was performed separately for every replicate (i.e., each dilution/optoplot) and the stated values represent mean ± SD of the average slope values. Background correction for fluorescence measurements was performed by subtracting the mean blank value (i.e., HS-167 in buffer only) from samples containing HS-167 and bacteria. Prism 8 (GraphPad Software Inc, USA) was used to plot the data and perform statistical tests. To compare the mean values of two different groups two-tailed *t*-test was used. When multiple *t*-tests were performed, we used false discovery rate (FDR) method of Benjamini, Krieger, and Yekutiele with desired FDR (*Q*) = 1%. To compare the mean values of several different groups one-way ANOVA was used. Tukey’s multiple comparisons test was used when all groups were compared to each other. Dunnett’s multiple comparisons test was used when all groups were compared to a control group. *F* and *p* values are stated in relevant figure captions. To compare the normalized slopes of Tn mutants to the control (WT, value of 100), we performed a one-sample *t*-test for each mutant applying a multiplicity adjusted *α* = 0.0001. Only mutants showing ≥25% difference from the WT were considered biologically significant.

### Reporting summary

Further information on experimental design is available in the Nature Research Reporting Summary linked to this paper.

## Supplementary information

Supplementary Information

Reporting Summary

## Data Availability

The authors declare that all data supporting the findings of this study are available within the paper and its supplementary information files.

## Code availability

The authors declare that the custom computer code is available upon request.
