# Author Correction: Optotracing for selective fluorescence-based detection, visualization and quantification of live *S. aureus* in real-time

**DOI:** 10.1038/s41522-020-00179-z

**Published:** 2021-01-08

**Authors:** Karen Butina, Ana Tomac, Ferdinand X. Choong, Hamid Shirani, K. Peter R. Nilsson, Susanne Löffler, Agneta Richter-Dahlfors

**Affiliations:** 1 AIMES-Center for the Advancement of Integrated Medical and Engineering Sciences at Karolinska Institutet and KTH Royal Institute of Technology, Stockholm, Sweden; 2 Department of Neuroscience, Karolinska Institutet, SE-171 77 Stockholm, Sweden; 3 Department of Chemistry, IFM, Linköping University, SE-581 83 Linköping, Sweden

Correction to: *npj Biofilms Microbiomes* 10.1038/s41522-020-00150-y, published online 9 October 2020

The original version of the published Article contained an error in the “Methods” section and the flow chart depicted in Fig. 3. The statistical test used should be a one sample *t*-test, and the alpha level 0.05. This has been corrected in the HTML and PDF version of the Article.

