# Supplementary material for: Optotracing for selective fluorescence-based detection, visualization and quantification of live S. aureus in real-time
Source: NPJ Biofilms Microbiomes. 2020 Oct 9;6:35. doi: 10.1038/s41522-020-00150-y (PMC7547713; doi:10.1038/s41522-020-00150-y)
Supplement: Supplementary file 1 — Supplementary Information [file 41522_2020_150_MOESM1_ESM.pdf]

SUPPLEMENTARY INFORMATION

Optotracing for selective fluorescence-based detection, visualisation and quantification of live *S. aureus* in real-time

Karen Butina[1,2], Ana Tomac[1,2], Ferdinand X. Choong[1,2], Hamid Shirani[3], K. Peter R. Nilsson[1,3], Susanne Löffler[1,2], Agneta Richter-Dahlfors[1,2,*]

[1] AIMES - Center for the Advancement of Integrated Medical and Engineering Sciences at Karolinska Institutet and KTH Royal Institute of Technology

[2] Department of Neuroscience, Karolinska Institutet, SE-171 77, Stockholm, Sweden

[3] Department of Chemistry, IFM, Linköping University, SE-581 83, Linköping, Sweden

* Correspondence to Agneta Richter-Dahlfors: agneta.richter.dahlfors@ki.se

Page index

Figure S1 *Fluorescence spectroscopy and microscopy using optotracers*…………………….............1

Figure S2 *Buffered TSB prevents acidification of the bacterial culture*……………………..……2 - 3

Figure S3 *Automated growth curve analysis* ………………………………………………........4 - 6

Figure S4 *Growth of S. aureus USA300 JE2 in bTSB ± HS-167*……………………...…………......7

Figure S5 *High-throughput screening of the Tn library*…………………..………………………8 – 11

Figure S6 *Optotracing of E. faecalis*……………………………………… …………..……........12

Figure S7 *Effect of buffer conditions on spectra and fluorescence intensity of HS-167*...............12 – 13

Table S1 *Bacterial strains*……………………..……………………...……………………..…......14

Table S2 *Transposon mutants with slopes significantly lower than WT USA300 JE2*………..…15 – 17

Supplementary Figure 1

Fluorescence spectroscopy and microscopy using optotracers

(a, b) Normalized spec-plots of **a)** HS-84 and **b)** HS-163 added to *S. aureus* (magenta) and *S.* Enteritidis (grey) in PBS, and to PBS only as control (dotted black). Lines show mean values of n = 3. **(c-e)** Merged transmitted light and pseudocolored confocal images of *S. aureus* (left) and *S.* Enteritidis (right) mixed with **c)** HS-84 (green), **d)** HS-163 (cyan), **e)** HS-167 (magenta), in PBS. Scale bar = 10 μm. Images are collected at excitation and emission wavelengths as indicated in each panel. White boxes indicate areas shown enlarged in **Fig. 1b, c, d**, respectively.

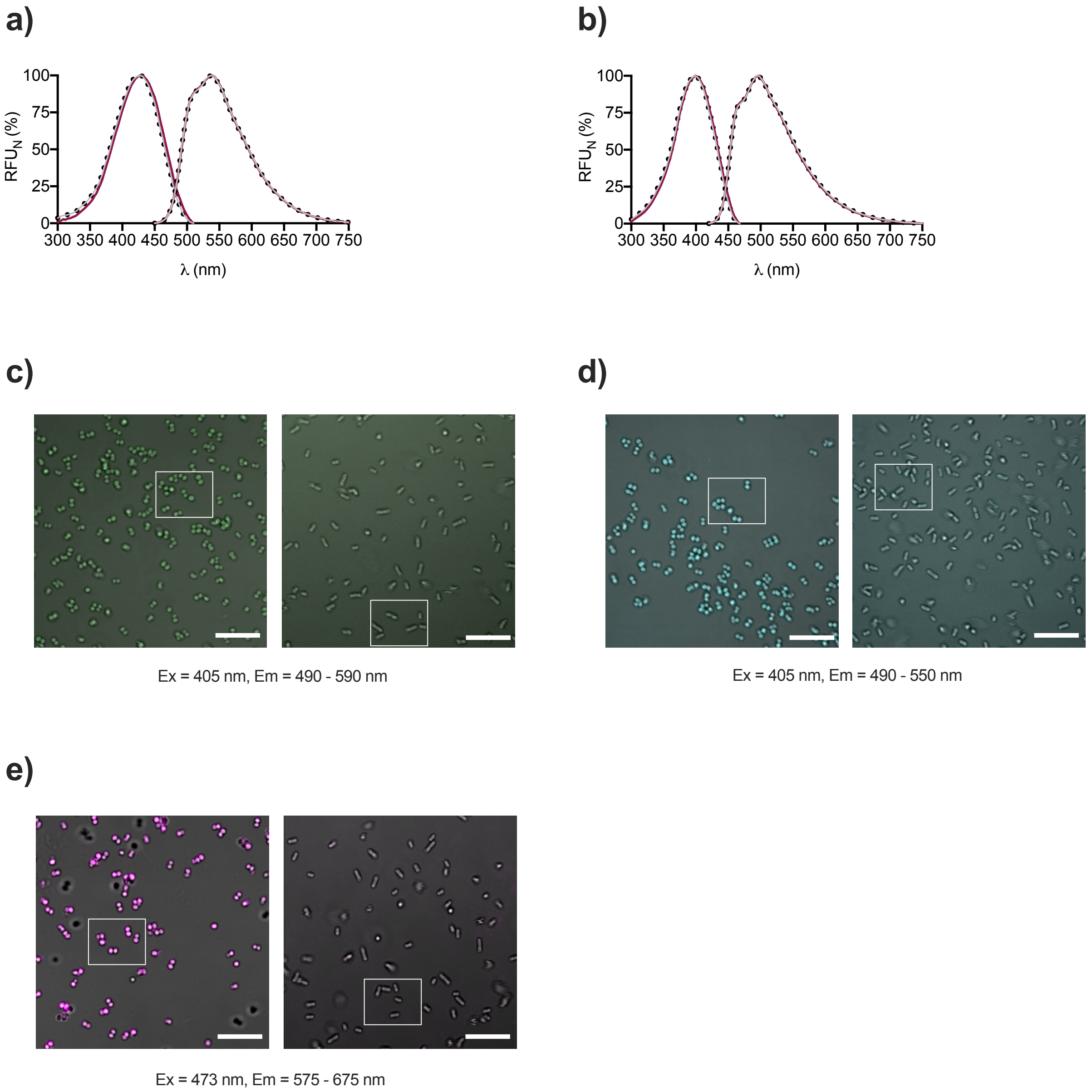

Supplementary Figure 2

Buffered TSB prevents acidification of the bacterial culture

a) Fluorescence intensity recorded as relative fluorescence units (RFU) at Ex. 507 nm and Em. 625 nm of HS-167 in TSB pH 7.1 and TSB adjusted to pH 5.1. n = 3, error bars = SD. (**b-d)** Growth of *S. aureus* in TSB (black) and bTSB (grey). **b)** The pH of the culture at different OD_{600} (n = 3). **c)** OD_{600} of the culture over time (n = 3). **d)** Colony forming units (CFU) in the culture at different OD_{600} (n = 2). Symbols represent data points, with squares, triangles and filled circles each representing an individual experiment. **e)** pH dependent relative macrospecies distribution of HS-167, calculated using Chemicalize, developed by ChemAxon.

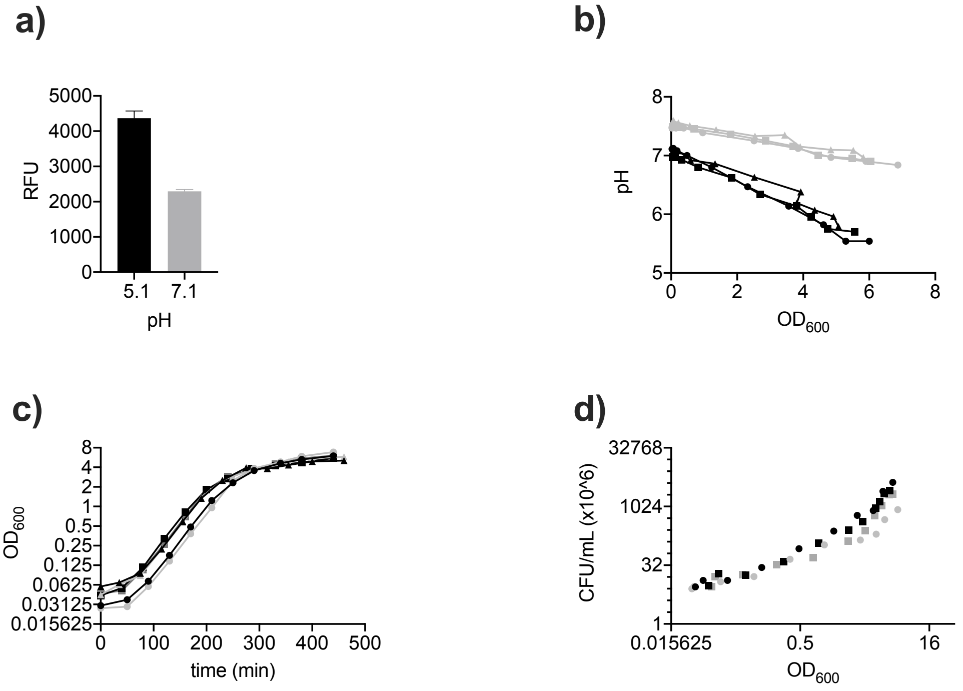

e)

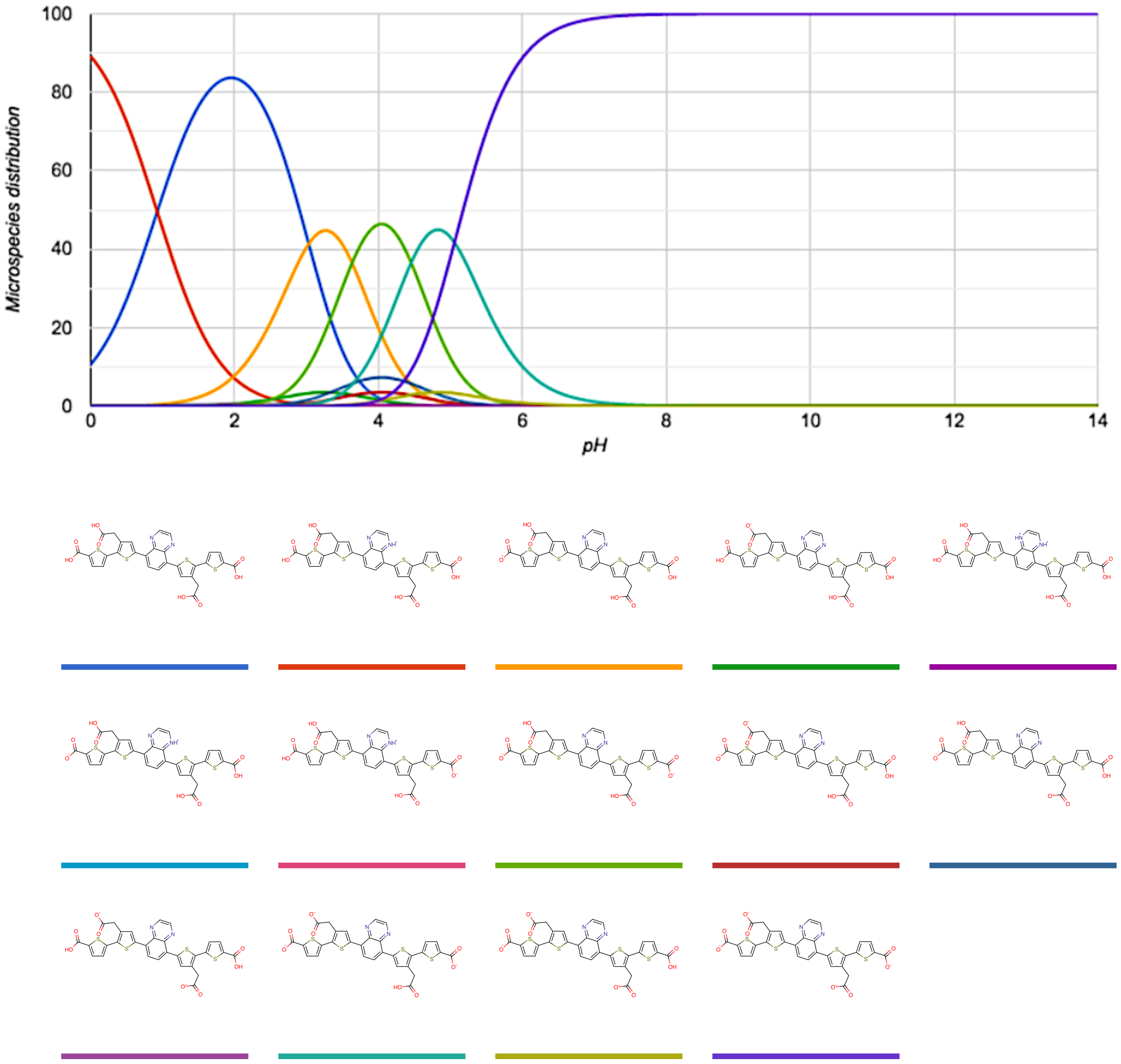

Supplementary Figure 3

Automated growth curve analysis

Analysis of growth curves of **a)** *S. aureus*, **b)** *S. epidermidis* and **c)** *S.* Enteritidis in bTSB ± HS-167.

Yellow line = mean A_{600} of technical triplicates for each experimental triplicate.

Green line = Savitzky-Golay filtered A_{600} (left Y axis).

Purple line = the gradient of filtered A_{600} (right Y axis).

Black dotted line = time frame of exponential growth, defined by top 20 % of gradient values.

a)

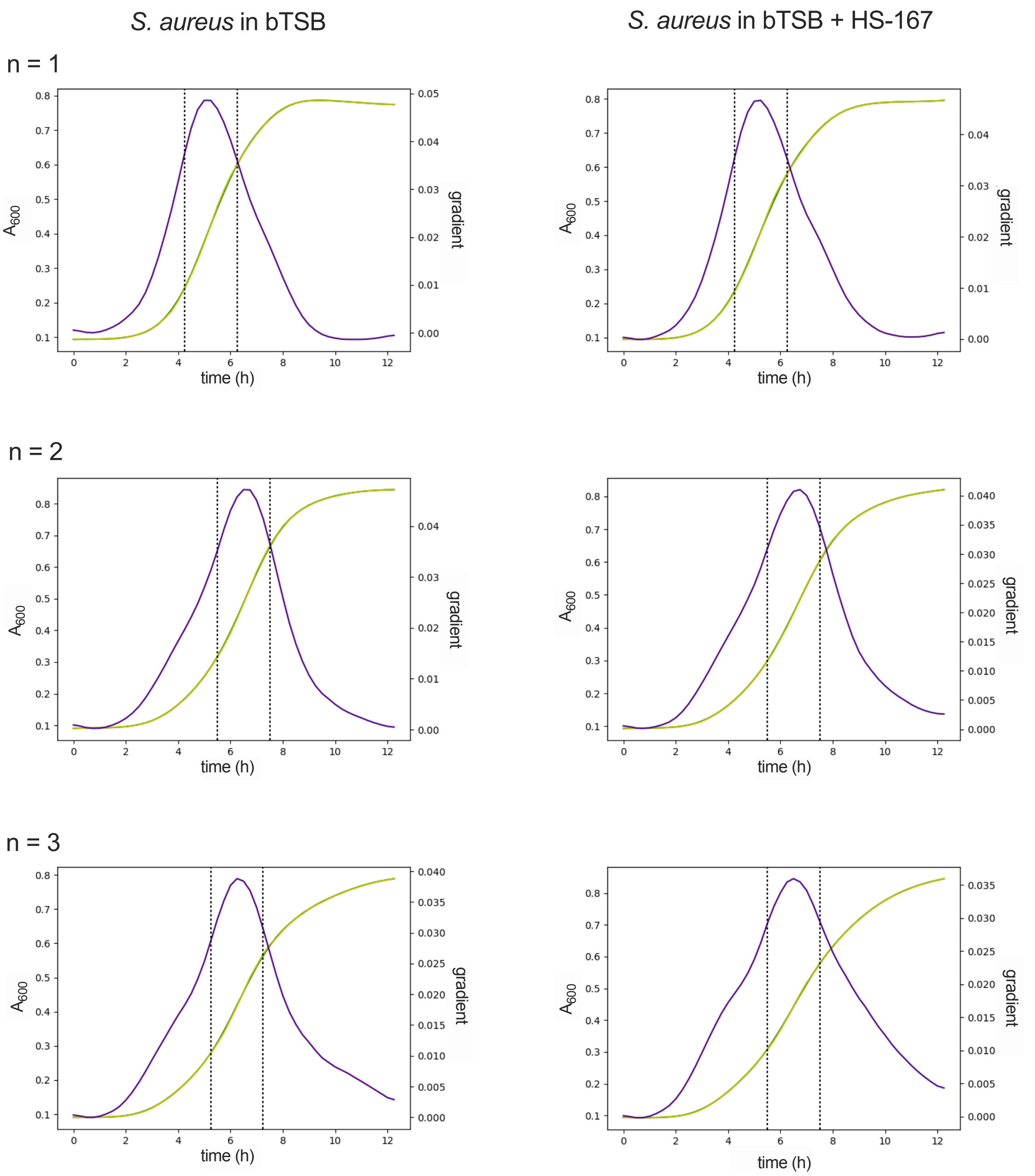

b)

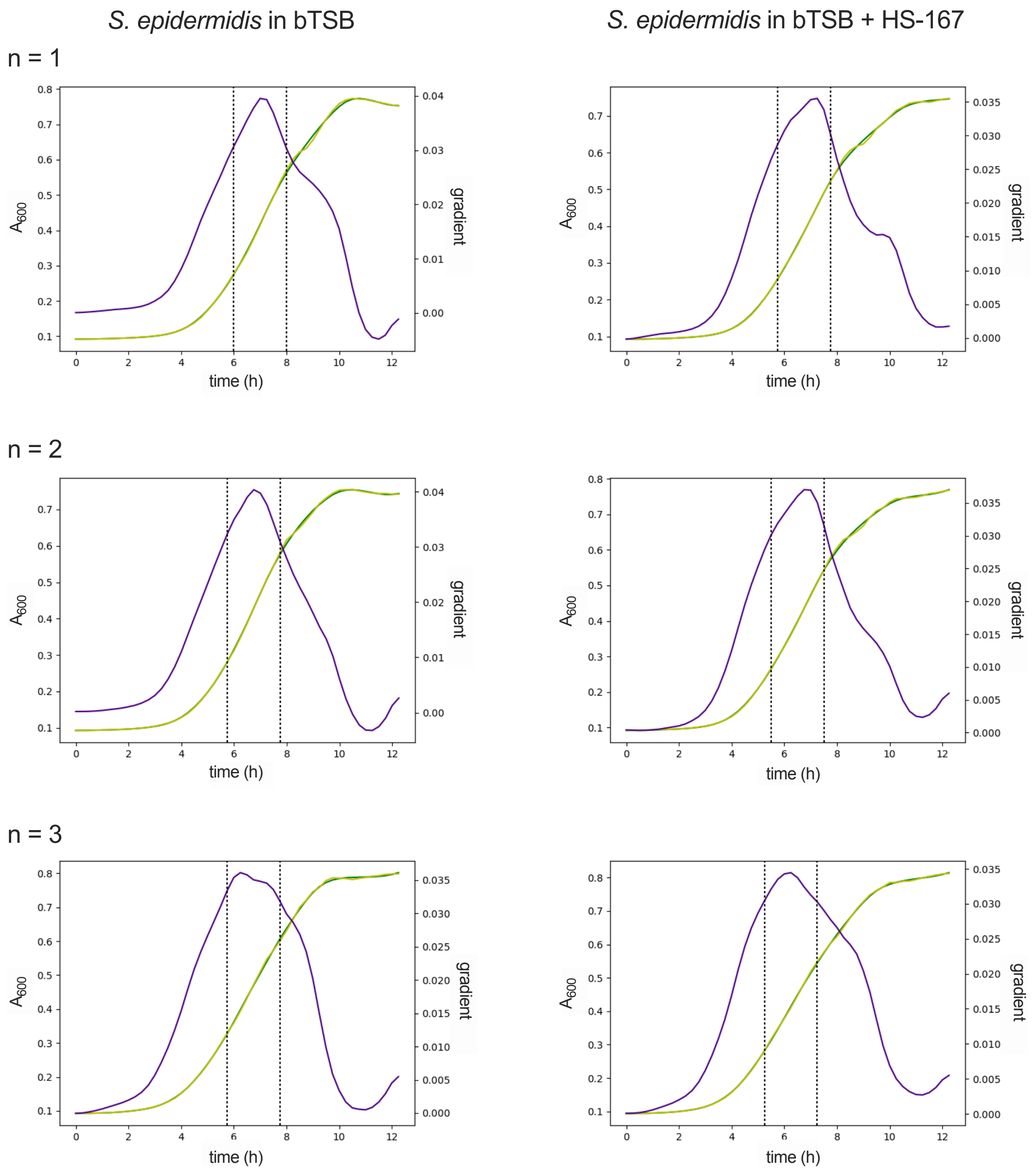

S. epidermidis in bTSB
S. epidermidis in bTSB + HS-167
n = 1
n = 2
n = 3
A600
gradient
time (h)

c)

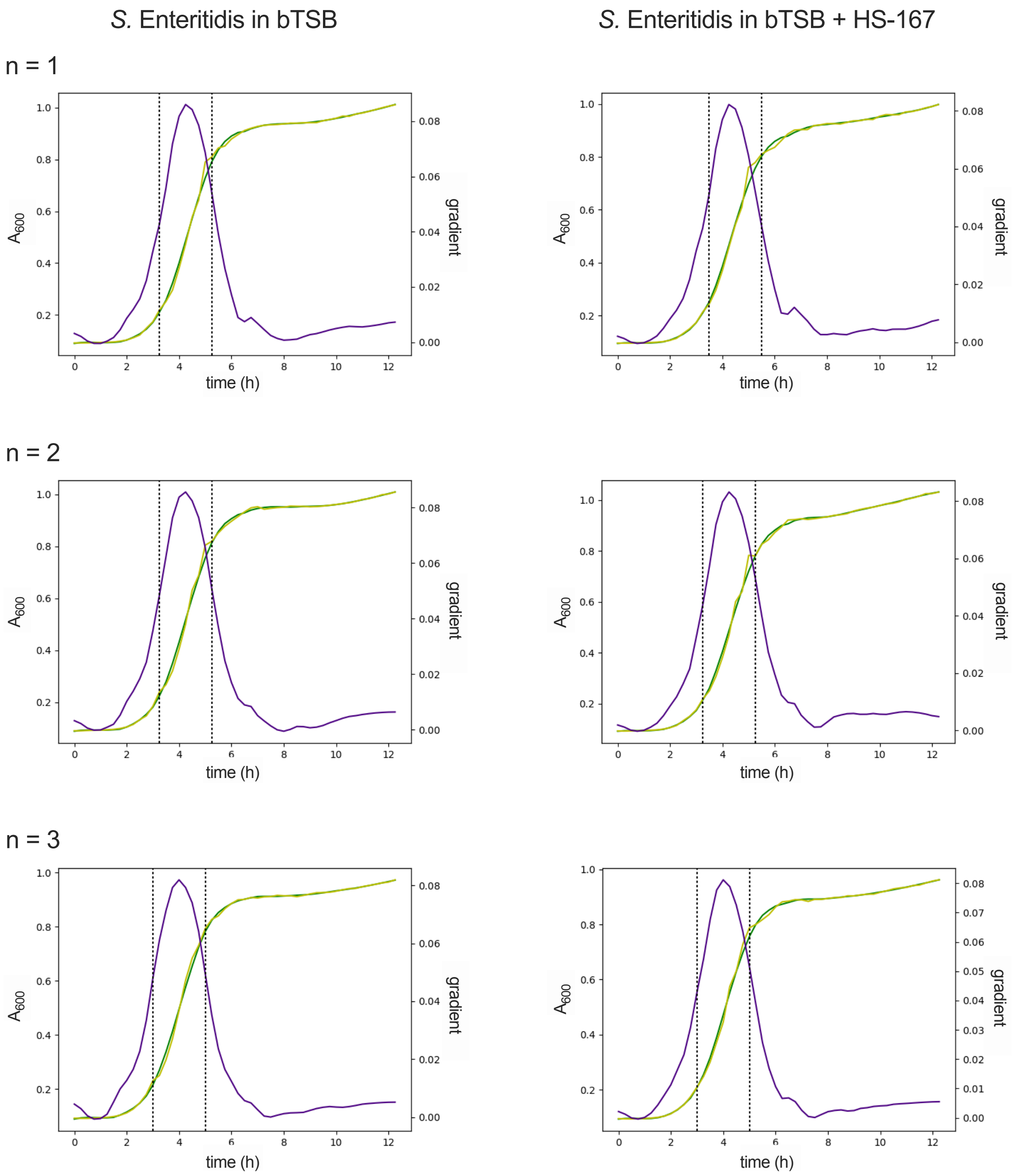

S. Enteritidis in bTSB
S. Enteritidis in bTSB + HS-167
n = 1
n = 2
n = 3
A_{600}
gradient
time (h)

Supplementary Figure 4

Growth of *S. aureus* USA300 JE2 in bTSB ± HS-167

(a-c) Growth of *S. aureus* USA300 JE2 in TSB (black) and bTSB (grey). **a)** The pH of the culture at different OD_{600}, **b)** OD_{600} of the culture over time, and **c)** colony forming units (CFU) in the culture at different OD_{600} . Symbols represent data points, n = 1. **d)** Generation time of *S. aureus* USA300 JE2 grown in 96 well plates in filtered TSB (TSB), bTSB and bTSB + HS-167, and autoclaved TSB. n = 6, bars = mean + SD.

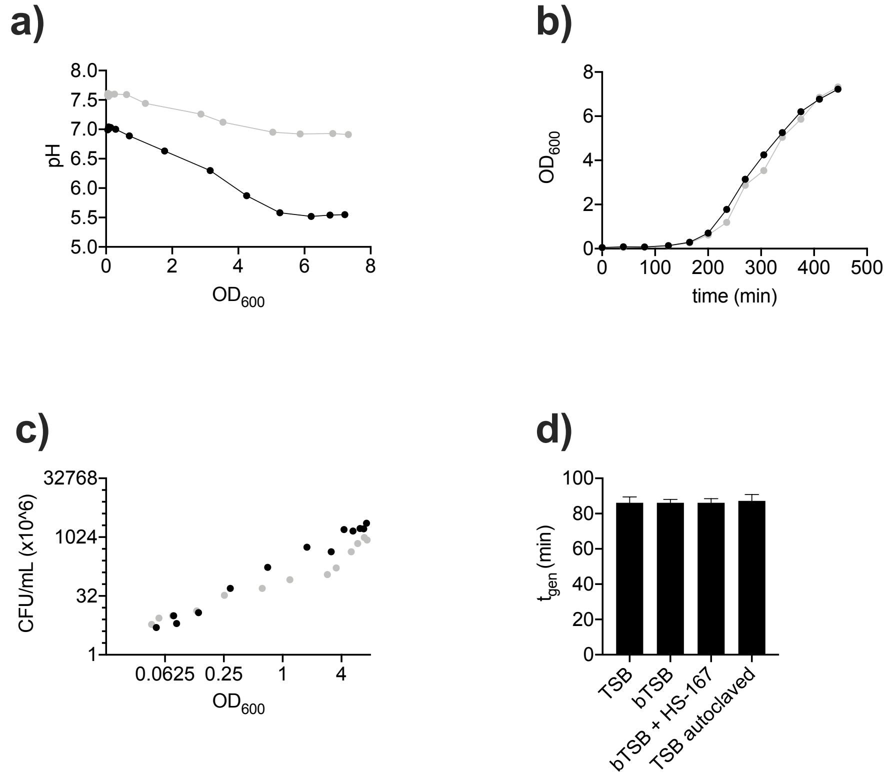

Supplementary Figure 5

High-throughput screening of the Tn library

a) Slopes of the 1917 mutants shown by heat map representation. Stars mark the selected candidate mutants, white crossed wells show excluded mutants. **b)** Slopes generated by optoplot analysis of the 95 selected Tn mutants (named by SAUSA300 codes) normalized to the slope of the WT strain USA300 JE2 (dotted line). n = 4, error bars = SD.

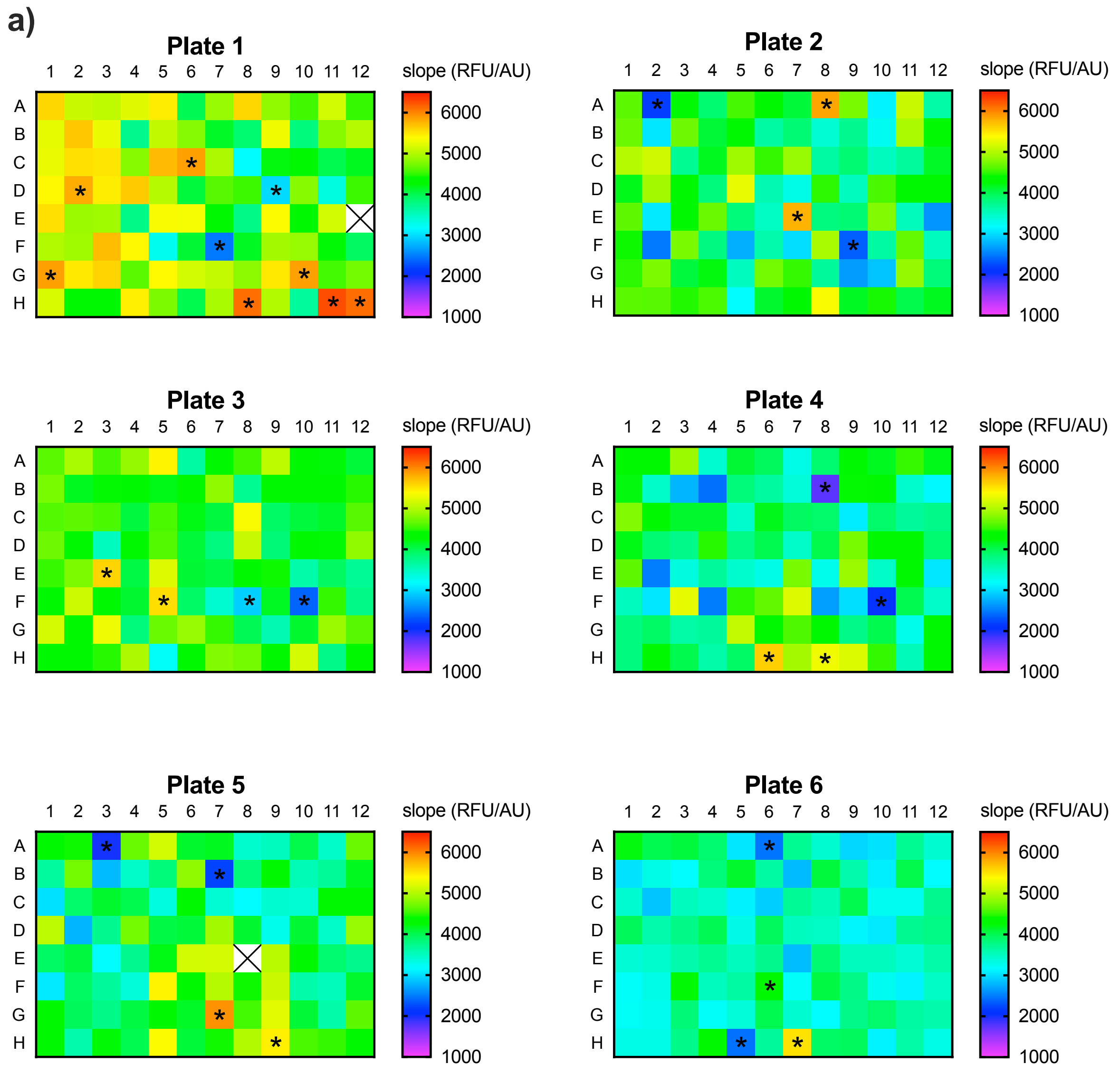

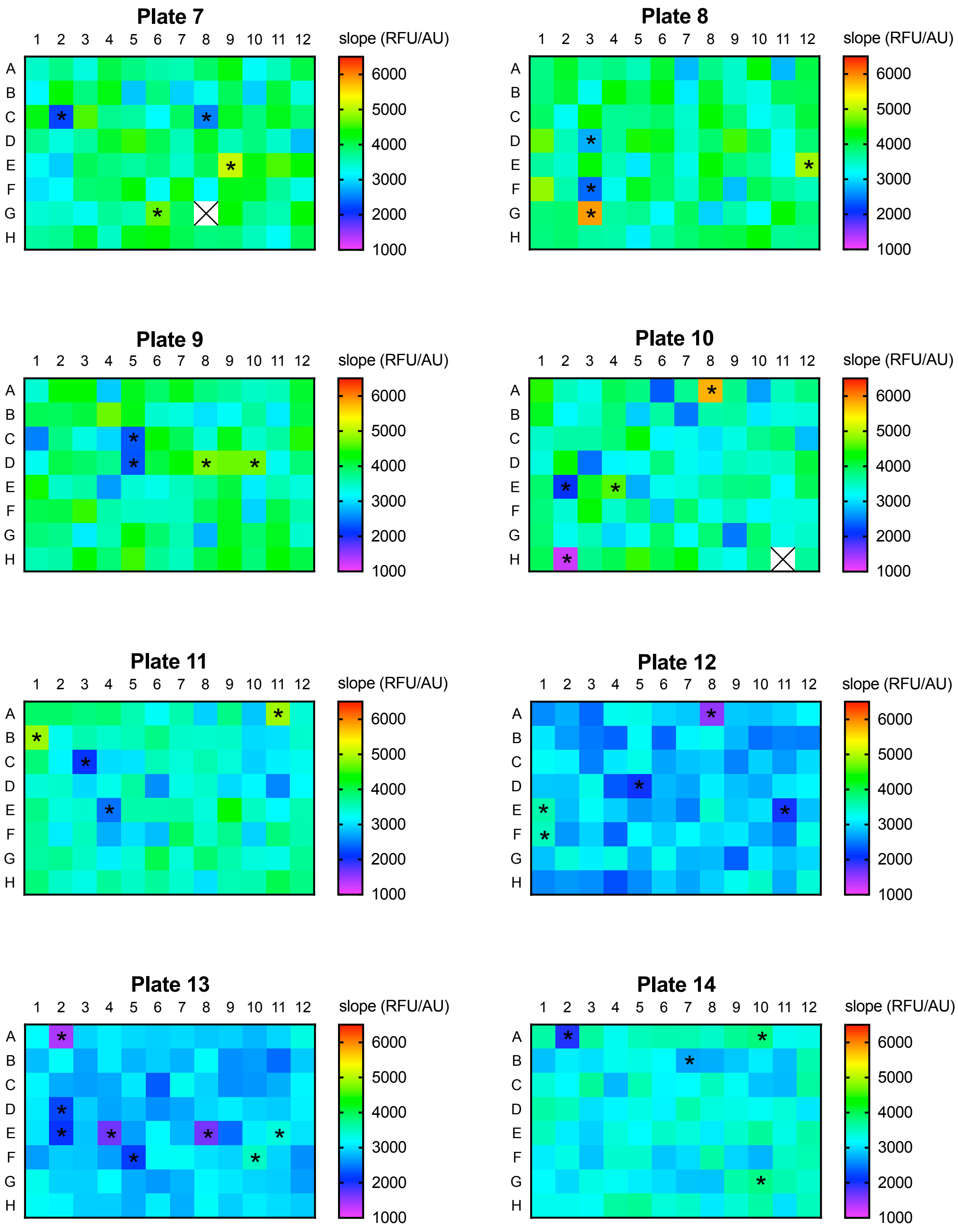
Plate 7
Plate 8
Plate 9
Plate 10
Plate 11
Plate 12
Plate 13
Plate 14
1 2 3 4 5 6 7 8 9 10 11 12
A B C D E F G H
slope (RFU/AU)
6000
5000
4000
3000
2000
1000

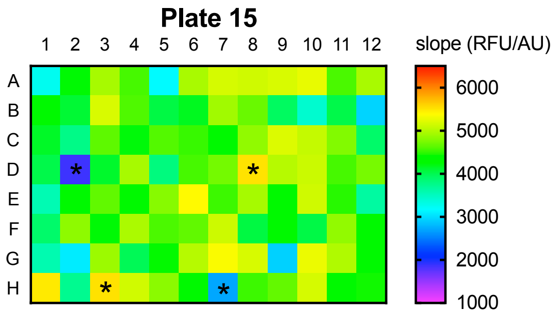
Plate 15
1 2 3 4 5 6 7 8 9 10 11 12
A B C D E F G H
slope (RFU/AU)
6000
5000
4000
3000
2000
1000

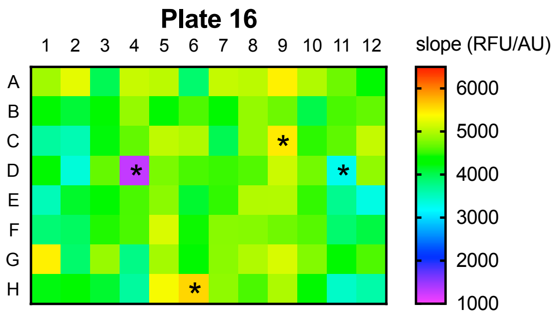
Plate 16
1 2 3 4 5 6 7 8 9 10 11 12
A B C D E F G H
slope (RFU/AU)
6000
5000
4000
3000
2000
1000

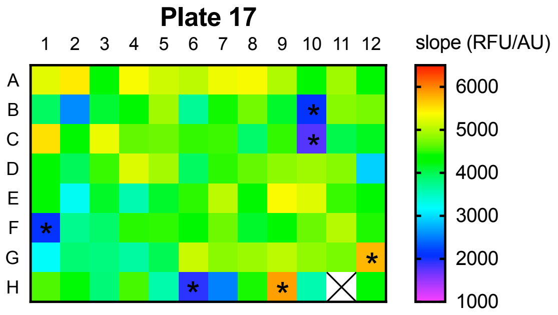
Plate 17
1 2 3 4 5 6 7 8 9 10 11 12
A B C D E F G H
slope (RFU/AU)
6000
5000
4000
3000
2000
1000

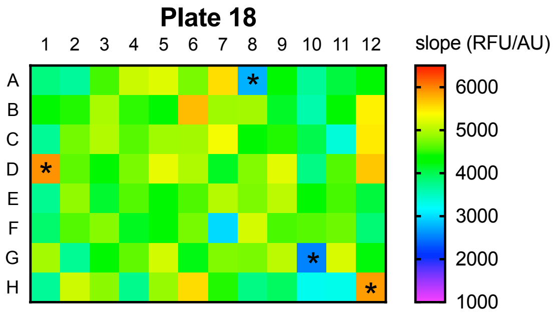
Plate 18
1 2 3 4 5 6 7 8 9 10 11 12
A B C D E F G H
slope (RFU/AU)
6000
5000
4000
3000
2000
1000

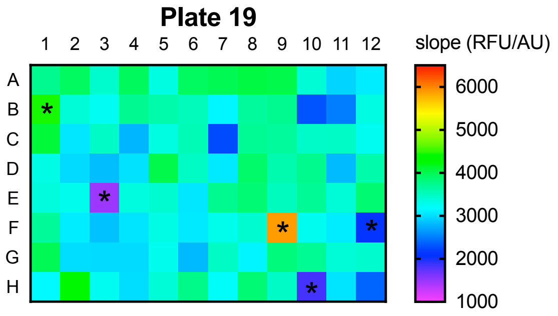
Plate 19
1 2 3 4 5 6 7 8 9 10 11 12
A B C D E F G H
slope (RFU/AU)
6000
5000
4000
3000
2000
1000

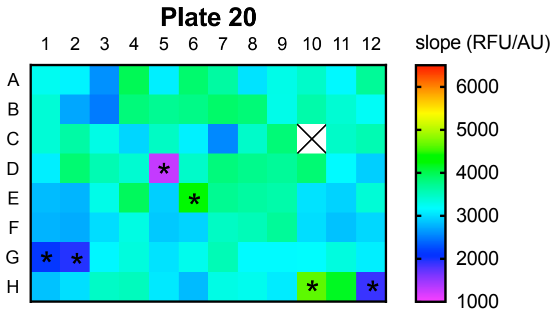
Plate 20
1 2 3 4 5 6 7 8 9 10 11 12
A B C D E F G H
slope (RFU/AU)
6000
5000
4000
3000
2000
1000

b)

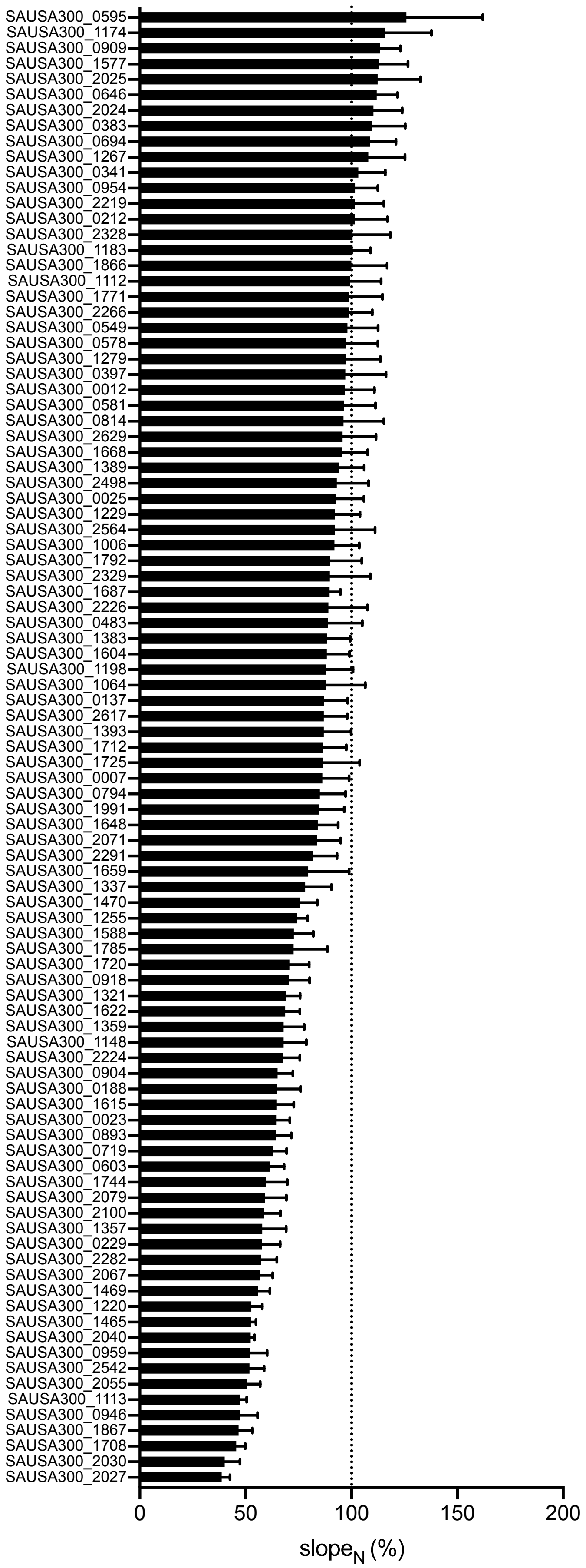
SAUSA300_0595
SAUSA300_1174
SAUSA300_0909
SAUSA300_1577
SAUSA300_2025
SAUSA300_0646
SAUSA300_2024
SAUSA300_0383
SAUSA300_0694
SAUSA300_1267
SAUSA300_0341
SAUSA300_0954
SAUSA300_2219
SAUSA300_0212
SAUSA300_2328
SAUSA300_1183
SAUSA300_1866
SAUSA300_1112
SAUSA300_1771
SAUSA300_2266
SAUSA300_0549
SAUSA300_0578
SAUSA300_1279
SAUSA300_0397
SAUSA300_0012
SAUSA300_0581
SAUSA300_0814
SAUSA300_2629
SAUSA300_1668
SAUSA300_1389
SAUSA300_2498
SAUSA300_0025
SAUSA300_1229
SAUSA300_2564
SAUSA300_1006
SAUSA300_1792
SAUSA300_2329
SAUSA300_1687
SAUSA300_2226
SAUSA300_0483
SAUSA300_1383
SAUSA300_1604
SAUSA300_1198
SAUSA300_1064
SAUSA300_0137
SAUSA300_2617
SAUSA300_1393
SAUSA300_1712
SAUSA300_1725
SAUSA300_0007
SAUSA300_0794
SAUSA300_1991
SAUSA300_1648
SAUSA300_2071
SAUSA300_2291
SAUSA300_1659
SAUSA300_1337
SAUSA300_1470
SAUSA300_1255
SAUSA300_1588
SAUSA300_1785
SAUSA300_1720
SAUSA300_0918
SAUSA300_1321
SAUSA300_1622
SAUSA300_1359
SAUSA300_1148
SAUSA300_2224
SAUSA300_0904
SAUSA300_0188
SAUSA300_1615
SAUSA300_0023
SAUSA300_0893
SAUSA300_0719
SAUSA300_0603
SAUSA300_1744
SAUSA300_2079
SAUSA300_2100
SAUSA300_1357
SAUSA300_0229
SAUSA300_2282
SAUSA300_2067
SAUSA300_1469
SAUSA300_1220
SAUSA300_1465
SAUSA300_2040
SAUSA300_0959
SAUSA300_2542
SAUSA300_2055
SAUSA300_1113
SAUSA300_0946
SAUSA300_1867
SAUSA300_1708
SAUSA300_2030
SAUSA300_2027
0
50
100
150
200
slope_N (%)

Supplementary Figure 6

Optotracing of *E. faecalis*

a) Generation time for *E. faecalis* grown in 96 well plates in bTSB with (grey) and without (black) HS-167. Lines show mean ± SD from n = 3. **b)** Spec-plots of HS-167 added to *E. faecalis* (black line) and to PBS only as control (dotted black line). Lines show mean values of n = 5, shaded areas show ± SD.

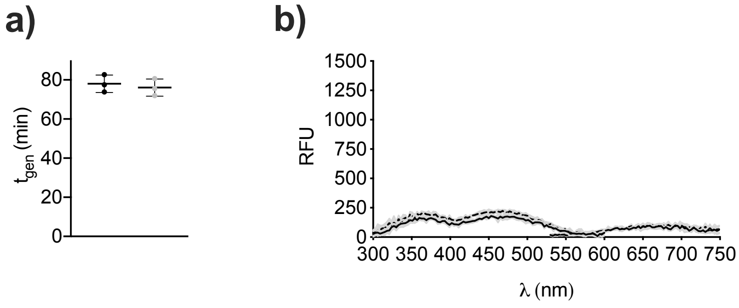

Supplementary Figure 7

Effect of buffer conditions on spectra and fluorescence intensity of HS-167

To determine the effect of pH and ionic strength on the fluorescence intensity, excitation spectrum and emission spectrum of unbound HS-167, we prepared buffers ranging from pH 3.0 - 9.0 with 0, 150 and 500 mM NaCl. To span the wide pH range, we used 20 mM di-sodium phosphate/citric acid (pH 3.0, 5.0, 7.0) or 20 mM Tris/hydrochloric acid (pH 7.0, 9.0).

Fig. S7a shows fluorescence intensity (RFU) of HS-167 (Ex. 507 nm, Em. 625 nm) in different buffer conditions. n = 3, error bars = SD. Fluorescence intensity was higher at acidic pH than at neutral and basic pH. At pH 3.0, we observed significantly reduced solubility of HS-167, presumably due to attenuation of electrostatic repulsion as a consequence of protonation. Highest fluorescence intensity was observed at pH 5.0, probably caused by the balanced contribution of π-stacking between the aromatic rings which enabled intermolecular electron transfer, and hydrogen bonding between the protonated and non-protonated carboxyl groups. HS-167 at pH 7.0 and pH 9.0 showed similar fluorescence intensity, as it adopts same conformation at both pHs (see **Fig. S2e**). No major difference was observed between the two buffer systems (di-sodium phosphate/citric acid and Tris/hydrochloric acid buffers), showing that no specific interaction with buffer ions occurred that would influence fluorescence intensity. Addition of NaCl lead to an increased fluorescence intensity in buffers at pH 5.0, 7.0, and 9.0. At pH 3.0, the effect of increased hydrophobic interactions due to increased ionic strength was masked due to reduced solubility and aggregation at this pH.

Fig. S7 b-d shows how the spectral characteristics of HS-167 are influenced by different pHs and salt concentrations. Using the same buffer systems as in (**a**), spectra of HS-167 at different pHs (color coded as in (**a**)) in the presence of **b)** 0 mM NaCl, **c)** 150 mM NaCl and **d)** 500 mM NaCl were recorded and are presented as spec-plots (left) and normalized spec-plots (right). Lines show mean values from n = 3. Irrespective of NaCl, a general picture emerged showing that acidic pH caused red shifts in the excitation spectra, possibly because of protonation-induced planarization and stacking of HS-167, while the Stokes shift was smaller at pH 5.0, compared to pH 3.0. Moreover, at acidic pH, the excitation peak at longer wavelengths (peak 2) was higher than the excitation peak at shorter wavelengths (peak 1), while their intensities were similar for pH ≥ 7.0. As peak 1 represents π-π*

transition, and peak 2 represents D-A transition, their relative heights represent the nature of intramolecular electron transfer. The increase of NaCl to 150 mM and 500 mM had most pronounced effects on the spectrum at pH 5.0 (violet lines in panel b-d). The fluorescence intensity increased with increasing salt concentration and the ratios between the two excitation peaks (peak 2/peak 1) varied depending on the salt concentration (1.35 at 0 mM; 1.61 at 150 mM; 1.16 at 500 mM), suggesting that the use of 500 mM NaCl in the optotracing assay promotes π-π* transition in HS-167 at pH 5.0.

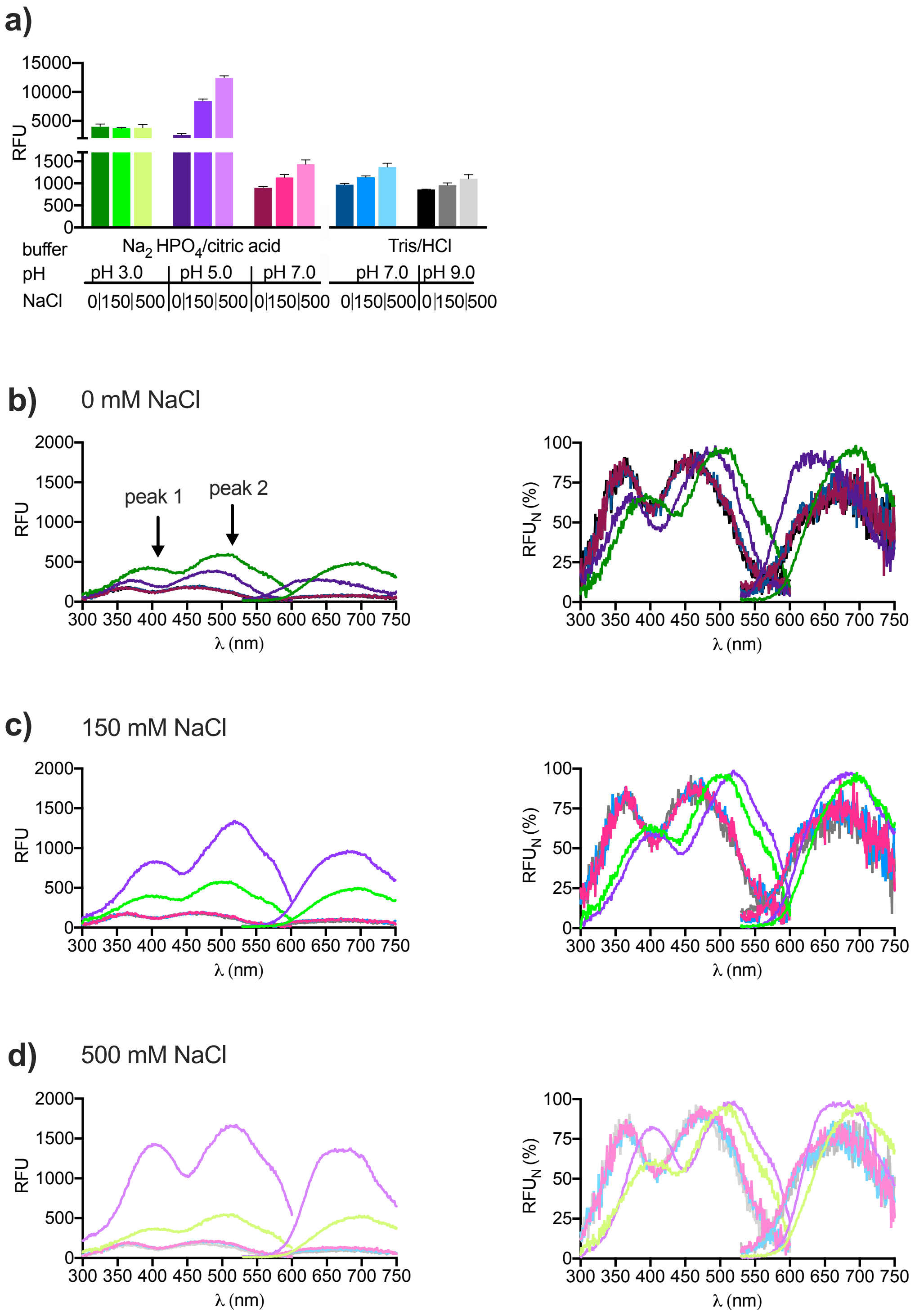

Supplementary Table 1: Bacterial strains

**Species and strains**	**Genotypic and phenotypic characteristics**	**Reference**
Salmonella Enteritidis 3934	clinical isolate	([1])
Staphylococcus epidermidis RP62A, ATCC 35984	clinical isolate	American Type Culture Collection (Germany)
Enterococcus faecalis ATCC 29212		American Type Culture Collection (Germany)
Staphylococcus aureus strains		
8325-4	Wild type strain, cured of prophages, ΔrsbU.	([2], [3])
8325-4 Δ*clpX*	Growth defect at 30° C, smaller colonies at 37°C.	([3])
8325-4 Δ*clpX* $ltaS_{382STOP}$	Does not grow at 44°C	([3])
USA300 JE2	*S. aureus* USA300 LAC cured of plasmids	([4])
SAUSA300_2055 (ΔmurA)		([4])
SAUSA300_2027 (Δalr)		([4])

References:

1. Solano, C. *et al*. Genetic analysis of Salmonella enteritidis biofilm formation: critical role of cellulose. *Mol. Microbiol.* **43**, 793–808 (2002).
2. Horsburgh, M. J. *et al*. sigmaB modulates virulence determinant expression and stress resistance: characterization of a functional rsbU strain derived from Staphylococcus aureus 8325-4. *J Bacteriol* **184**, 5457–5467 (2002).
3. Bæk, K. T. *et al*. The Cell Wall Polymer Lipoteichoic Acid Becomes Nonessential in Staphylococcus aureus Cells Lacking the ClpX Chaperone. *MBio* **7**, (2016).
4. Fey, P. D. *et al*. A Genetic Resource for Rapid and Comprehensive Phenotype Screening of Nonessential Staphylococcus aureus Genes. *MBio* **4**, (2013).

Supplementary Table 2 (complementary to **Fig. 3b**)

Transposon mutants with slopes significantly lower than WT USA300 JE2

**(SAUSA300_) [gene]**	**Function of deleted gene**
	**Cell envelope**
1255 [ΔfmtC]	A membrane anchored protein that affects methicillin resistance, (might be) associated with cell wall biosynthesis in the lipid cycle[5]. Catalyzes the transfer of a lysyl group from L-lysyl-tRNA(Lys) to membrane-bound phosphatidylglycerol, producing lysylphosphatidylglycerol, that is a major component of the bacterial membrane and has a positive net charge[6].
1588 [ΔlytH]	N-acetylmuramoyl-L-alanine amidase activity[6]. Controls cell size and division[7].
1785	Multidrug ABC transporter ATP-binding protein[8]. Mutant has decreased resistance towards polymyxins[9].
0918 [ΔugtP]	Also known as YpfP. Biosynthesis of bilayer- and non-bilayer-forming membrane glucolipids. Catalyzes the formation of a glycolipids used as a membrane anchor for LTA[6].<br>Mutants have decreased amount of phosphatidylcholine in cell membrane[10]. ugtP affects LTA structure[11] and reduces its content in some strains[12].
1622 [Δtig]	Chaperone activity. Involved in protein export. Functions as a peptidyl-prolyl cis-trans isomerase[6].
1359	Polyprenyl synthase, involved in menaquinone synthesis[13].
1615 [ΔhemB]	Heam synthesis, associated with SCVs[14] and increased resistance to animonglycosides[14,15].
0719 [ΔsstB]	Uptake of Fe(III)-catechol siderophores. sstA and sstB are hydrophobic membrane proteins that form a heterodimeric permease[16,17].
2100	Lytic regulatory protein[6].
1357 [ΔaroC]	Chorismate is a precursor for menaquinone biosynthesis. Mutation leads to reduced susceptibility to gentamicin due to altered membrane potential[15].
2282 [ΔlyrA]	Lysostaphin resistance protein A. Mutants are more resistant to lysostaphin but does not affect resistance to beta lactams[18]. Mutants have small amounts of truncated cross bridges or cross bridges with aberrant structure and a slight decrease in PGN crosslinking[18].
2040	Cell division protein FtsW[8]. FtsW is a peptidoglycan polymerase that requires divalent cat-ions and the presence of a class A penicillin binding proteins for activity[19].
0959 [Δfmt]	Beta lactamase activity[6]. Prevents autolysis and promotes antibiotic resistance[20].
2055 [ΔmurA]	Catalysing the first step of PGN biosynthesis[21].
1113 [ΔpknB]	Also known at Stk1[6]. Resistance to beta lactams[22,23]. Regulation of cell division and cell wall homeostasis[23]. Regulates TA alanylation[23].
0946 [ΔmenD]	Menaquinone synthesis, associated with SCVs[14] and increased resistance to animonglycosides[14,15].
1708 [Δrot]	Rot promotes expression of surface proteins[24].
2027 [Δalr]	Interconversion of D-alanine and L-alanine. D-alanine is present in PGN and D-alanine esters

	on TA modulate surface charge[25].
	**Amino acid synthesis, transport, metabolism and degradation**
0893 [ΔoppF]	AA transport and metabolism, regulated by CodY[6].
2067 [ΔglyA]	Catalyzes the reversible interconversion of serine and glycine and biosynthesis of other important molecules[6].
1465	Degradation of branched chain AA[6].
1469 [ΔargR]	Regulates arginine biosynthesis genes[6]**.**
	**Central carbon metabolism**
2079 [Δfba]	Fructose bisphosphate aldolase[6].
0229	Putative acyl-CoA transferase FadX[6].
	**Major regulators**
1148 [ΔcodY]	Regulates approx. 5 % of the *S. aureus* genome, majority of targets are repressed[26]. Regulation of AA biosynthesis, metabolism and transport, virulence associated genes[27–29] including the agr locus and saeRS two-component system[26].
1113 [ΔpknB]	Positive regulator of sigB[22].
1708 [Δrot]	Global regulator of virulence and biofilm associated genes. Regulated by sigB and agr[24,30].
	**Other or not annotated (N/A)**
1720	N/A
1321	N/A
2224 [ΔmoeA]	Molybdopterin biosynthesis protein[6].
0904	N/A
1188 [ΔmutS]	DNA mismatch repair protein[6].
0023	N/A
0603	N/A
1744	N/A
1220	LuxR family DNA-binding response regulator[6].
2542	Putative AMP-binding enzyme[6].
1867 [ΔvraT]	N/A
2030	N/A

References

5. Komatsuzawa, H. *et al*. Cloning and sequencing of the gene, fmtC , which affects oxacillin resistance in methicillin-resistant Staphylococcus aureus. *FEMS Microbiol. Lett.* **203**, 49–54 (2001).
6. The UniProt Consortium. UniProt: a worldwide hub of protein knowledge. *Nucleic Acids Res.* **47**, (2019).
7. Do, T. *et al*. Staphylococcus aureus cell growth and division are regulated by an amidase that trims peptides from uncrosslinked peptidoglycan. *Nat. Microbiol.* **5**, 291–303 (2020).
8. Fuchs, S. *et al*. Aureo Wiki‑The repository of the Staphylococcus aureus research and annotation community. *Int. J. Med. Microbiol.* **308**, 558–568 (2018).
9. Vestergaard, M. *et al*. Inhibition of the ATP Synthase Eliminates the Intrinsic Resistance of Staphylococcus aureus towards Polymyxins. *MBio* **8**, (2017).
10. Ayala, O. D. *et al*. Drug-Resistant Staphylococcus aureus Strains Reveal Distinct Biochemical Features with Raman Microspectroscopy. *ACS Infect. Dis.* **4**, 1197–1210 (2018).
11. Gründling, A. & Schneewind, O. Genes Required for Glycolipid Synthesis and Lipoteichoic Acid Anchoring in Staphylococcus aureus. *J. Bacteriol.* **189**, 2521–2530 (2007).
12. Fedtke, I. *et al*. A Staphylococcus aureus ypfP mutant with strongly reduced lipoteichoic acid (LTA) content: LTA governs bacterial surface properties and autolysin activity. *Mol. Microbiol.* **65**, 1078–1091 (2007).
13. Desai, J. *et al*. Structure, Function, and Inhibition of Staphylococcus aureus Heptaprenyl Diphosphate Synthase. *ChemMedChem* **11**, 1915–1923 (2016).
14. Proctor, R. A. *et al*. Small colony variants: A pathogenic form of bacteria that facilitates persistent and recurrent infections. *Nature Reviews Microbiology* **4**, 295–305 (2006).
15. Vestergaard, M., Nøhr-Meldgaard, K. & Ingmer, H. Multiple pathways towards reduced membrane potential and concomitant reduction in aminoglycoside susceptibility in Staphylococcus aureus. *Int. J. Antimicrob. Agents* **51**, 132–135 (2018).
16. Morrissey, J. A., Cockayne, A., Hill, P. J. & Williams, P. *Molecular Cloning and Analysis of a Putative Siderophore ABC Transporter from Staphylococcus aureus. Infection and immunity* **68**, (2000).
17. Conroy, B. S., Grigg, J. C., Kolesnikov, M., Morales, D. L. & Murphy, M. E. P. Staphylococcus aureus heme and siderophore-iron acquisition pathways. *BioMetals* **32**, 409–424 (2019).
18. Gründling, A., Missiakas, D. M. & Schneewind, O. Staphylococcus aureus Mutants with Increased Lysostaphin Resistance. *J. Bacteriol.* **188**, 6286–6297 (2006).
19. Taguchi, A. *et al*. FtsW is a peptidoglycan polymerase that is functional only in complex with its cognate penicillin-binding protein. *Nat. Microbiol.* **4**, 587–594 (2019).
20. Utaida, S. *et al*. Genome-wide transcriptional profiling of the response of Staphylococcus aureus to cell-wall-active antibiotics reveals a cell-wall-stress stimulon. *Microbiology* **149**, 2719–2732 (2003).
21. Blake, K. L. *et al*. The nature of Staphylococcus aureus MurA and MurZ and approaches for detection of peptidoglycan biosynthesis inhibitors. *Mol. Microbiol.* **72**, 335–343 (2009).
22. Tamber, S., Schwartzman, J. & Cheung, A. L. Role of PknB kinase in antibiotic resistance and virulence in community-acquired methicillin-resistant Staphylococcus aureus strain USA300. *Infect. Immun.* **78**, 3637–3646 (2010).
23. Pensinger, D. A., Schaenzer, A. J. & Sauer, J.-D. Do Shoot the Messenger: PASTA Kinases as Virulence Determinants and Antibiotic Targets Phosphorylation in Bacterial Pathogens. *Trends Microbiol* **26**, 56–69 (2018).
24. Mootz, J. M. *et al*. Rot is a key regulator of Staphylococcus aureus biofilm formation. *Mol. Microbiol.* **96**, 388–404 (2015).
25. Moscoso, M., Garc Ia, P., Cabral, M. P., Rumbo, C. & An Bou, G. A D-Alanine auxotrophic live vaccine is effective against lethal infection caused by Staphylococcus aureus. *Virulence* **9**, 604–620 (2018).
26. Kaiser, J. C. & Heinrichs, D. E. Branching Out: Alterations in Bacterial Physiology and Virulence Due to Branched-Chain Amino Acid Deprivation. *MBio* **9**, (2018).
27. Kaiser, J. C., Omer, S., Sheldon, J. R., Welch, I. & Heinrichs, D. E. Role of BrnQ1 and BrnQ2 in Branched-Chain Amino Acid Transport and Virulence in Staphylococcus aureus. *Infect. Immun.* **83**, 1019 –1029 (2015).
28. Pohl, K. *et al*. CodY in Staphylococcus aureus: A regulatory link between metabolism and virulence gene expression. *J. Bacteriol.* **191**, 2953–2963 (2009).
29. Majerczyk, C. D. *et al*. Direct targets of CodY in Staphylococcus aureus. *J. Bacteriol.* **192**, 2861–2877 (2010).
30. Hsieh, H.-Y., Wen Tseng, C. & Stewart, G. C. Regulation of Rot Expression in Staphylococcus aureus. *J. Bacteriol.* **190**, 546–554 (2008).
